# Leveraging Blood Components for 3D Printing Applications Through Programmable Ink Engineering Approaches

**DOI:** 10.1002/advs.202406569

**Published:** 2024-10-25

**Authors:** Rita Sobreiro‐Almeida, Sara C. Santos, Monize C. Decarli, Marcelo Costa, Tiago R. Correia, Joanna Babilotte, Catarina A. Custódio, Lorenzo Moroni, João F. Mano

**Affiliations:** ^1^ Department of Chemistry CICECO – Aveiro Institute of Materials University of Aveiro Campus Universitário de Santiago Aveiro 3810‐193 Portugal; ^2^ Complex Tissue Regeneration department MERLN Institute for Technology‐Inspired Regenerative Medicine Maastricht University Universiteitssingel 40 Maastricht 6229 ET The Netherlands; ^3^ Metatissue – PCI Creative Science Park Aveiro Region Ílhavo 3830‐352 Portugal

**Keywords:** 3D printing, albumin, hydrogel, ink engineering, photocrosslinking, platelet lysates, protein

## Abstract

This study proposes a tunable ink engineering methodology to allow 3D printing processability of highly bioactive but otherwise low‐viscous and unprintable blood‐derived materials. The hypothesis relies on improving the viscoelasticity and shear thinning behavior of platelet lysates (PL) and albumins (BSA) solutions by covalent coupling, enabling simultaneous extrusion and photocrosslinking upon filament deposition. The available amine groups on proteins (PL and BSA) are exploited for coupling with carboxyl groups present in methacrylated proteins (hPLMA and BSAMA), by leveraging carbodiimide chemistry. This reaction enabled the creation of a pre‐gel from these extremely low‐viscous materials (≈ 1 Pa), with precise tuning of the reaction, resulting in inks with a range of controlled viscosities and elasticities. Shape‐fidelity analysis is performed on 3D‐printed multilayered constructs, demonstrating the ability to reach clinically relevant sizes (>2 cm in size). After photocrosslinking, the scaffolds showcased a mechanically robust structure with sustained protein release over time. Bioactivity is evaluated using human adipose‐derived stem cells, resulting in increased viability and metabolic activity over time. The herein described research methodology widens the possibilities for the use of low‐viscosity materials in 3D printing but also enables the direct application of patient and blood‐derived materials in precision medicine.

## Graphical abstract

Patient‐derived materials show great potential for tissue engineering applications. Nevertheless, they usually lack the necessary properties for additive manufacturing. To address this, an innovative approach based on protein chemical coupling is developed to improve rheological properties of low‐viscous blood‐derived components. The improvement of viscoelasticity and shear‐thinning behavior of platelet lysates and albumins allows for 3D printing of robust and customized matrices.

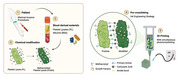

## 1 Introduction

Protein‐based materials, particularly those sourced from human proteins such as extracellular matrices (ECM), have been increasingly explored for tissue engineering and regenerative medicine applications.^[^ ^1^, ^2^ ^]^ Their intrinsic biocompatibility and richness in bioactive proteins involved in cell adhesion, maintenance, and growth are essential features for bioapplicability. Among them, blood‐derived resources have recently gained the spotlight, as these materials can have an autologous origin, and are notably rich in essential bioactive domains and growth factors.^[^ ^3^ ^]^ Albumin, for instance, has been explored as an attractive biomaterial for biomedical research and therapeutics due to its versatility and availability.^[^ ^4^ ^]^ Being the most abundant plasma protein and readily obtainable, albumin‐based scaffolds have demonstrated potential for clinical applicability, as coating methods for implants,^[^ ^5^ ^]^ as platforms for in vitro cell culture,^[^ ^6^ ^]^ or even as biocompatible resins for use in stereolithography (SLA) printing, when modified with photocurable moieties (BSAMA).^[^ ^7^ ^]^ Another promising blood‐derived component is human platelet lysates (PL). PL are easily obtained from platelet‐rich plasma (PRP) and have been used in cell culture as a replacement for animal‐derived supplements, addressing concerns, such as ethical issues and the risk of transmitting xenogeneic contaminants.^[^ ^8^ ^]^ Besides their allogenic or autologous origin, PL comprises a source of bioactive proteins and growth factors essential for cell maintenance and proliferation.^[^ ^1^, ^9^ ^]^ Human methacryloyl platelet lysates (hPLMA) have been proposed as a novel photocrosslinkable precursor material to produce mechanically tunable matrices with reported applications on stem cell culture,^[^ ^10^ ^]^ and 3D cancer models.^[^ ^11^ ^]^ Although PL and albumin can be seen as valuable materials for bioengineering/biofabrication purposes, they lack the required rheological properties for extrusion 3D printing, demonstrating an extremely low viscous nature, which limits their applicability in this field.

Extrusion 3D printing is based on the fabrication of structures in a layer‐by‐layer manner leading to the development of customized constructs with precise control over the spatial and structural properties. An ideal ink for extrusion 3D printing should present specific physicochemical properties such as high viscosity, shear‐thinning, and elastic recovery,^[^ ^12^, ^13^ ^]^ which cannot be accomplished on blood‐derived components, as previously stated. In order to overcome their lack of suitable rheological properties – mandatory for the extrusion printing process –, chemical modification of these polymers (with light‐sensitive moieties for instance),^[^ ^14^, ^15^ ^]^ or the combination with other polymeric matrices^[^ ^16^, ^17^, ^18^, ^19^ ^]^ have been the most attractive strategies for printing these materials. Other interesting and cutting‐edge strategies that have been put forward by researchers in recent years for printing low‐viscous matrices comprise the development of support baths,^[^ ^20^, ^21^, ^22^, ^23^ ^]^ aqueous two‐phase systems,^[^ ^24^ ^]^ polyelectrolyte complexation,^[^ ^25^ ^]^ and cryobioprinting,^[^ ^26^ ^]^ among others. Ink engineering approaches emerged in an attempt to reach higher viscosity states, making low‐viscous solutions suitable for extrusion printing. By controlling the materials on a molecular level, it is possible to tune specific properties, for example, shear‐thinning behavior or stimuli responsiveness, ultimately leading to the development of printable inks.^[^ ^3^, ^27^ ^]^

As such, we herein hypothesize a bioengineering approach for developing injectable materials or inks suitable for additive manufacturing processes, exclusively derived from blood components, such as albumin and PL. We thoroughly describe and characterize, for the first time, their rheological and printability features, with focus on the development of biologically relevant constructs suitable for tissue engineering purposes.

## 2 Results and Discussion

### 2.1 Ink Engineering Strategy

**Figure 1:**
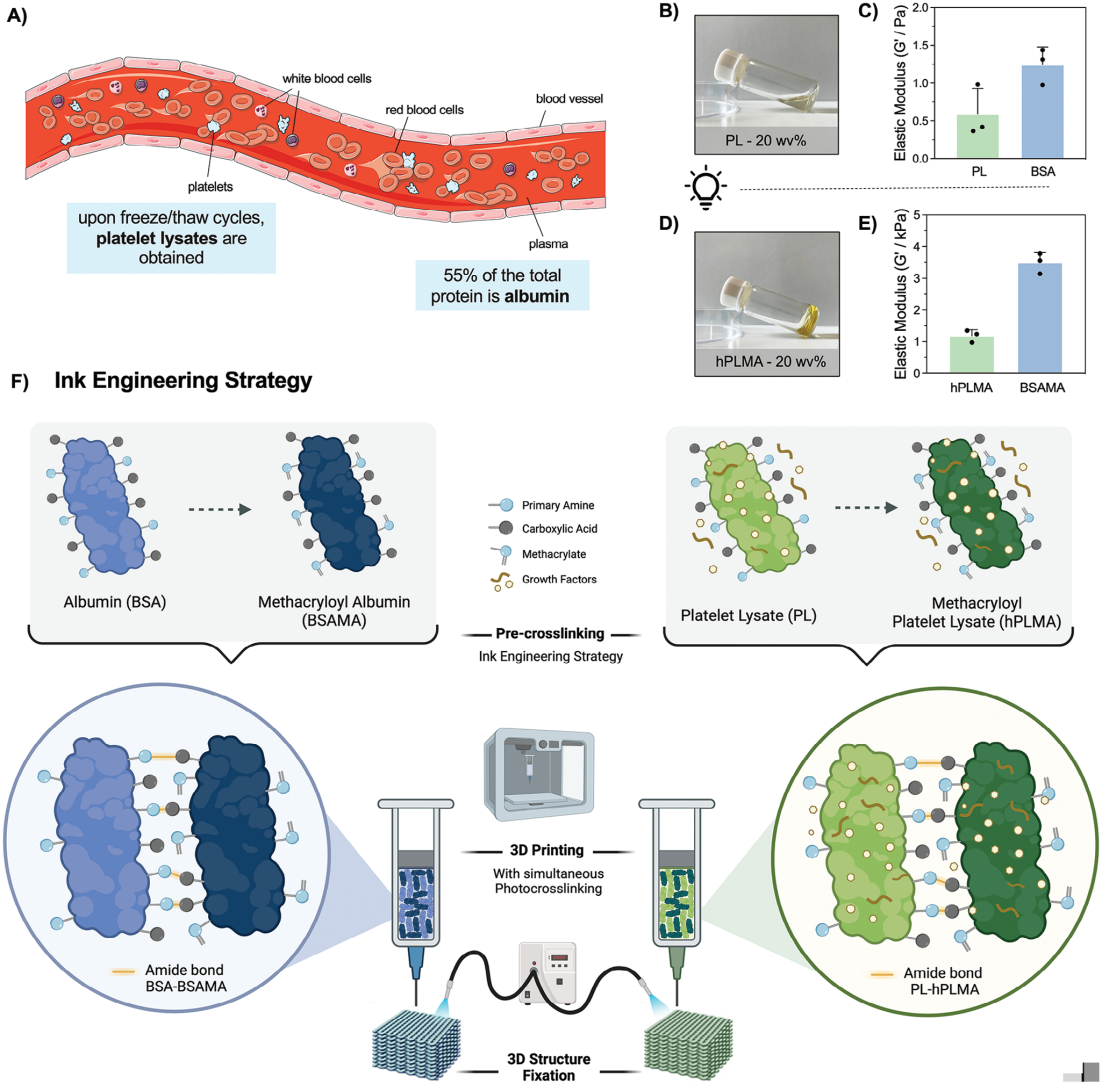
Rationale for the ink engineering approach. A) Representative scheme of a blood vessel in which the proposed ink components albumin (BSA) and platelet lysates (PL) are represented; B) Visual aspect of the solubilized PL at 20 wv% (BSA has the same liquid nature); C) Elastic moduli of the solutions of PL and BSA, at 20 and 10 wv%, respectively, demonstrating G’ ≈ 1 Pa, not suitable for 3D extrusion printing (*n* = 3); D) Visual aspect of the hPLMA solution after 60 s of photocrosslinking (again representative of hPLMA and BSAMA); E) Elastic moduli of the hPLMA and BSAMA solutions after photocrosslinking (1‐3 kPa, also not suitable for extrusion 3D printing) (*n* = 3); F) ink engineering hypothesis: coordinate the non‐functionalized protein with the methacrylated protein by carbodiimide chemistry, creating a pre‐crosslinking ink that upon extrusion can be photocrosslinked to fix the desired shape.

This work describes a bioengineering approach to obtain inks for 3D extrusion printing technology using byproducts derived from blood. Herein, platelet lysates (PL) from human blood and albumin from bovine serum (BSA) will be used as protein pools or model globular proteins, to demonstrate how to turn extremely low viscous matrices into higher viscous solutions, suitable for applications in additive manufacturing technologies (**Figure** **1**A). Due to their extremely low viscous nature (< 2 Pa elastic moduli, Figure 1B,C), they have been previously suggested for biomedical applications through chemical modification with photocurable moieties, rendering hydrogels with tunable stiffnesses (Figure 1D,E).^[^ ^10^, ^11^, ^28^ ^]^ Methacrylation was herein confirmed by ^1^H‐NMR (Figure S1, Supporting Information) and OPA colorimetric assay (Figure S2, Supporting Information). Although these hydrogels (BSAMA and hPLMA) have demonstrated promising applications as platforms for 3D cell culture and in vitro models,^[^ ^29^, ^30^ ^]^ their rheological properties did not meet the 3D extrusion printing criteria for layer‐by‐layer deposition.

For this purpose, we selected a carbodiimide cross‐linker (EDC/NHS) due to its recognized biocompatibility, reaction extent tunability, high water solubility, and mild reaction conditions.^[^ ^31^ ^]^ Additionally, by using the EDC and NHS mixture, not only the stability and efficiency of the reaction is enhanced, but also “zero‐length” amide bonds between carboxylic and primary amine groups are promoted, resulting in the production of urea as the sole byproduct that can be easily removed.^[^ ^32^ ^]^ Thus, to optimize the reaction conditions, we first examined the interaction of the methacrylated proteins alone by testing several protein concentrations and ratios of EDC:NHS (Table S1, Supporting Information). As this reaction is more prone to occur at lower pH, we also evaluate the impact of conducting the reaction in PBS (pH = 7.4) or MES buffer (pH = 5.5) (Table S2, Supporting Information). Among the results, the reversion of the EDC/NHS reaction due to hydrolysis, the precipitation of proteins and the use of high concentrations of cross‐linker were some of the major concerns (Figure S3, Supporting Information). To mitigate these, we combined – by zero‐length bonds – both the protein in its native and methacrylated form (Table S3, Supporting Information), obtaining promising results as no protein precipitation and no apparent hydrolysis occurred (Figure S4, Supporting Information). We were able to further reduce the concentration of EDC and NHS, by optimizing reaction conditions (Section 3, Figures S5 and S6, Supporting Information). The proposed reaction mechanism is schematized in Figure 1F. We hypothesize that the resulting pre‐cross‐linked material could be used as inks for 3D printing, taking advantage of the remaining methacryloyl groups to fix the final shape of the structure using light.

### 2.2 Rheological Characterization

**Figure 2:**
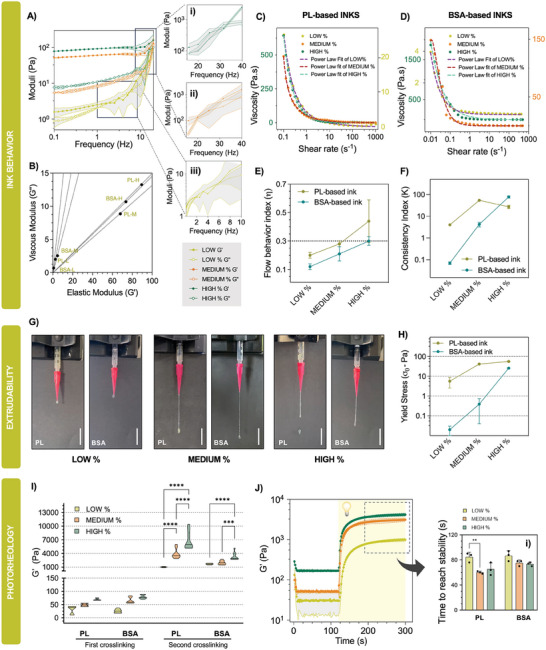
Rheological characterization of BSA and PL‐based inks with varying percentages of cross‐linker. A) Frequency sweeps of the inks at 0.1% strain (*n* = 3). Elastic and viscous moduli crossover points’ frequencies increased along with the concentration of cross‐linker. Zoom‐in of the crossover points for i) HIGH, ii) MEDIUM and iii) LOW%. B) Plotted loss tangent values obtained through strain sweep. Medium concentrations are theoretically closer to the ideal rheological properties; C,D) Mean values of flow sweeps for PL and BSA‐based inks, respectively (*n *= 3). Shear viscosity decreases with increasing shear rate corroborating shear‐thinning behavior. Lines represent the power law fit for the whole range of the sweep. Axes are colored according to the datapoints. In PL‐based inks, the dark green axis are valid for HIGH% and MEDIUM%; E) Variation of the flow behavior index (η) with the cross‐linker concentrations (*n *= 3); F) Variation of the consistency index (K) with the cross‐linker concentrations (*n *= 3); G) Maximum filament length obtained by doing extrudability test on the inks using 25G conical nozzles; H) Variation of yield stress (σ_0_) with the cross‐linker concentrations (*n *= 3); I) Plot of the elastic moduli obtained before and after photocrosslinking, indicating a clear dependency of the moduli on the cross‐linker concentration used, demonstrating statistical difference on the hydrogels post photocrosslinking. 2way ANOVA with multiple comparisons was used to compare between LOW, MEDIUM and HIGH% of cross‐linker in the same ink (*n *= 3); J) Time sweep performed on PL‐based inks at 0.1% strain and 1 Hz frequency with light irradiation at t=120 s (*n *= 3); i) Calculation of the time to reach elastic moduli stability (< 20% variation in moduli) after photoreticulation for PL and BSA‐based inks. 2way ANOVA was used to compare the time to reach stability between LOW, MEDIUM and HIGH% of the same ink (*n *= 3). All data is expressed as means ± standard deviation (grey shading in plotted curves or error bars in column graphs), except for graphs C,D), in which only mean values are represented for clearer visualization of the results and fitted curves. ^****^ *p* < 0.001; ^***^ *p* < 0.005; ^**^ *p* < 0.01.

By varying cross‐linker concentrations in the pre‐crosslinking approach, the tunability of the proposed reaction to develop inks from blood components has further been demonstrated (Figures S5 and S6, Supporting Information). In order to gain a deeper insight into the flow behavior of these inks, their rheological properties, including photorheology, viscoelasticity, yield stress and extrudability, were thoroughly studied, by using three different concentrations of cross‐linker, named LOW%, MEDIUM% and HIGH% for both inks, corresponding to 2.0, 2.2, and 2.4 vv% EDC/NHS for PL‐based inks and 1.2, 1.3, and 1.4 vv% of EDC/NHS for BSA‐based inks, respectively (**Figure** **2**). These key rheological properties may be accurate predictors of the potential printing fidelity.

We first studied the inks by assessing their viscoelasticity under different frequencies (Figure 2A) and amplitudes (Figure S7, Supporting Information) of oscillation. The linear viscoelastic region (LVE) was determined to be below 1% strain for LOW%, MEDIUM% and HIGH% (Figure S7A,B, Supporting Information), and these values were applied in subsequent measurements. Interestingly, the behavior of all inks on strain dependent sweeps was found to be similar for both proteins. BSA and PL‐based inks displayed its yield point and flow point (G’’ > G’) above 10% and 100% strain values, respectively. The yield and crossover point was found to be highly relatable to the number of cross‐links or entanglements on the inks, with higher cross‐link density providing increasing resistance against change in shape and permanent deformation (G’’ > G’).^[^ ^33^ ^]^ Our results both in PL and BSA‐based inks corroborate these findings:, HIGH% and MEDIUM% inks display higher frequency (Figure 2A‐i,ii, respectively) S7, values for the crossover point, comparing with the LOW% (Figure 2A‐iii). Additionally, the elastic moduli (G’) has revealed to be dependent on the cross‐linker concentration in both rheological tests and in both protein inks, a finding that is corroborated by other studies in the literature.^[^ ^14^, ^34^ ^]^ By using storage and loss modulus values within LVE, we calculated the damping factor or loss tangent (tan(δ), Figure 2B and **Table** **1**), a value that identifies a suitable balance between flow and shape retention. Based on these values, a suitable classification of ink materials can be performed: lower loss tan(δ) indicates solid‐like behavior of materials while higher tan(δ) is indicative of a more liquid‐like behavior.^[^ ^33^, ^35^ ^]^ Our results demonstrate that lower tan(δ) values were found with the increase in cross‐linker concentration, with values ranging from 0.13 to 0.77. A good balance between filament uniformity and overall structural integrity of 3D printed constructs was reported for tan(δ) values between 0.24 and 0.45 in gelatin‐alginate inks,^[^ ^35^, ^36^ ^]^ and between 0.54 and 0.58 in tyramine hyaluronan inks,^[^ ^37^ ^]^ which indicates that tan(δ) values are highly dependent on the type of materials and concentrations used on the ink. Nevertheless, these studies also indicate that values below 0.2 (consistent with HIGH% formulations) are usually associated brittle and non‐printable inks, and values above 0.7 (consistent with LOW% formulations) can be extruded with ease but lack mechanical properties for shape retention after printing, which corroborates the liquid‐like behavior. These values suggest that the MEDIUM% formulation could be well‐suited for extrusion 3D printing.

**Table 1:** Viscosity profiles for PL and BSA‐based inks, with different cross‐linker concentrations. Shear‐thinning exponents (flow index, n and consistency index, K) were obtained through power law regression of the linear regions. The inks’ yield stress (σ_0_) was obtained through Herschel‐Bulkley model fitting to shear rate/shear stress sweeps. Shear rate at the wall ($\dot{\gamma }$) and loss tangent values (tan(δ)) – were calculated from flow indexes and amplitude sweeps, respectively (*n* = 3).

INK		Power Law			${\dot{\gamma }}_{wall}$ (s^−1^)	tan(δ)	Herschel‐Bulkley	
		*n*	K (Pa.s^n^)	R^2^			σ_0_ (Pa)	R^2^
**PL**	**LOW %**	0.2 ± 0.02	4.04 ± 0.17	0.99	1230.77	0.76	5.64 ± 3.10	0.99
	**MEDIUM %**	0.28 ± 0.02	54.07 ± 1.00	0.99	1010.99	0.20	41.55 ± 0.83	0.99
	**HIGH %**	0.44 ± 0.15	27.22 ± 4.66	0.72	811.19	0.15	56.38 ± 1.56	0.97
**BSA**	**LOW %**	0.12 ± 0.02	0.07 ± 0.01	0.99	1743.59	0.77	0.02 ± 0.02	0.99
	**MEDIUM %**	0.21 ± 0.05	4.23 ± 1.02	0.96	1194.14	0.53	0.39 ± 0.35	0.99
	**HIGH %**	0.3 ± 0.03	77.75 ± 9.58	0.99	974.36	0.14	25.47 ± 1.57	0.97

The measurement of the ink's viscosity under a range of shear rates can be useful for the prediction of printing fidelity and accuracy. Typically, higher viscosities lead to increased printing fidelity, but also lead to increased shear stress, which is not beneficial either for cell encapsulation, or for post‐printing elastic recovery. Herein, viscosity was measured as a function of shear rate in both PL‐ (Figure 2C) and BSA‐based inks (Figure 2D). Our findings demonstrate high drops in viscosity along with the application of higher shear rates in all inks, i.e., the inks demonstrate shear‐thinning behavior. This behavior is extremely important in 3D printing applications, as these conditions replicate the flow of the ink while passing through the nozzle, where the shear forces dramatically increase. Additionally, increasing viscosity values were observed as the concentrations of the cross‐linker augmented, both in PL‐ and BSA‐based inks. This behavior was expected, as the shear‐thinning ability of pre‐gelled solutions comes from polymer disentanglement and macromolecular orientation along the flow, which is offset by the cross‐linker concentration.^[^ ^33^ ^]^

To better predict optimal printing conditions, empirical models can be fit to shear viscosity data. Fitting curves and explanations of the fitting range can be found in Figure S8 (Supporting Information) for BSA‐based inks and Figure S9 (Supporting Information) for PL‐based inks. By applying the power law model to the linear region, empirical flow indexes (*n*) – or shear thinning indexes – can be retrieved (Figure 2E and Table 1). For shear‐thinning materials, the flow index is expected to be 0 < *n* < 1; however, an ink with lower *n* value is considered to have higher shear‐thinning properties.^[^ ^38^ ^]^ Our results demonstrate that all inks fall within the shear‐thinning range (Figure S9, Supporting Information). Additionally, the obtained *n* value icreased with the increasing concentration of the cross‐linker (Figure 2E and Table 1), demonstrating that higher concentrations of the cross‐linker do not favor shear‐thinning properties (*n* > 0.3). Consequently, we hypothesize that smooth printing conditions will not be obtained with these inks, i.e., the filament could easily break upon extrusion. Another value that can be retrieved from the power law is the consistency index (*K*), i.e., the predicted value of viscosity at 1 s^−1^ shear rate (Figure 2F and Table 1).^[^ ^39^ ^]^ Our results indicate that *K* increases along with the concentration of the cross‐linker, with one exception being the HIGH% for PL‐based inks, which value drops comparing to the MEDIUM%. This implies a higher viscosity drop on HIGH%, suggesting that low stress rates may increase viscous behavior on these inks. Nonetheless, it was difficult to fit the HIGH% values to the power law model (*R^2^* = 0.72), so major conclusions will not be drawn from these fitting predictions. Another model that takes into consideration additional aspects such as yield stress, shear response and even wall slippage – which power law fails to predict – is the Herschel‐Bulkley model (Figure 2H and Table 1). In practice, this model gives additional information on the minimum stress needed for initiating a flow: the yield stress (σ^0^). It is the most employed empirical model in hydrogel research, as it allows for a deeper look into the relationships between material properties and rheological performance – including variables for both yield stress and shear response.^[^ ^40^ ^]^ Corroborating previous results, the calculated values increased along with the cross‐linker concentration, all of them resulting below 100 Pa, a value in concordance with other hydrogel inks in the literature.^[^ ^41^ ^]^ Shape‐retention is also highly related to the yield stress of a material, being an extremely important parameter for further clinical and surgical translation.^[^ ^40^ ^]^

Although these models provide useful insights on the behavior of different materials or the addition of cross‐linkers when and after passing through a nozzle, they can also be misleading, as the measurements do not consider other aspects such as surface tension or dynamic viscoelastic properties.^[^ ^38^ ^]^ Thus, to further corroborate our results with experimental data, we did a preliminary extrusion test using a pump in order to evaluate the material's ability to form fibers, instead of droplets or irregular filaments. The pump extrusion parameters were ubiquitous for all conditions for a better comparison of results. Several needle and nozzle sizes were tested: conical 25G (Figure 2G), conical 27G and 22G as well as metallic 25G (Figures S10–S12, Supporting Information). Our results indicate that independently of the applied nozzle, the LOW% concentration is not able to form filaments in any of the conditions and corroborates the empirical results of the high loss tangent values. On the opposite site, the HIGH% only enabled the formation of filaments using conical nozzles – and not metallic ones – most probably due to the increasing shear and yield stress during extrusion, as predicted by the Herschel‐Buckley model. These filaments demonstrate irregular shapes, and break easily, one aspect that is also corroborated by the low loss tangent values and the higher flow index. The MEDIUM%, however, demonstrates the formation of uniform filaments in all nozzles, except for 22G which may not confer the necessary shear/yield stress for viscous flow to be obtained. Comparing with the remaining nozzles, the filaments formed on 25G nozzles are more uniform and demonstrate less breaks during extrusion. Additionally, in order to understand the relevant shear rates in 3D printing context, we calculated the maximum shear rate at the wall using previously described equations^[^ ^42^, ^43^ ^]^ and obtained values from 811 to 1743 s^−1^ (Table 1), with higher values observed for the lowest percentages of cross‐linker. Nevertheless, because conical nozzles were found to be more suitable for obtaining cohesive filaments in the extrudability test, lower shear rates are expected comparing to a standard blunt needle.^[^ ^44^ ^]^ Thus, we hypothesize that the obtained shear rate values for the MEDIUM% ink fall within linear shear‐thinning region on flow curves. We further predicted the shear rate in pipe flow occurring at the wall of the nozzle at different nozzles gauges and printing speeds (Table S4, Supporting Information), confirming that smaller needle diameters and higher printing speeds increase the shear rate at the wall. This enables an accurate prediction of how inks will behave during 3D extrusion printing, by crossmatching these values with the inks' flow behavior and the respective viscosity values. Thus, even though high shear rates are not recommended, for instance for cell encapsulation and bioinks, a sufficient shear rate value must be applied to the ink in order to have enough viscosity drop, flow behavior and smooth filament formation.

Finally, to simulate post‐printing conditions, we applied photorheology and evaluated the elastic moduli (G’) before and after photocrosslinking (Figure 2I), the photocrosslinking kinetics on PL and BSA‐based inks (Figure 2J; Figure S7C, Supporting Information, respectively) and the necessary exposure time for the inks to reach elastic moduli stability (Figure 2J‐i). The results confirmed a marked dependency of the modulus before and after photocrosslinking to the cross‐linker concentration used. Moreover, a good indication for 3D printing is that these gels, especially the MEDIUM% formulation reach moduli stability after 60 to 80 s of light irradiation, a crucial value for setting up the 3D printing conditions.

Overall, the combined results of the rheology testing with mathematical modelling indicate that there is an optimal cross‐linker concentration and an optimal nozzle size (and type) to move to the printing evaluation. Additionally, we were able to predict the behavior of these inks during printing by testing extrudability and by applying mathematical models to more than one nozzle size and printing velocity. This type of evaluation is essential to accelerate the transition from a material concept to a printable ink, allowing scientists to save time and better predict and understand the behavior of their lab‐developed inks.

### 2.3 Structural and Mechanical Properties of Photocrosslinked Scaffolds

**Figure 3:**
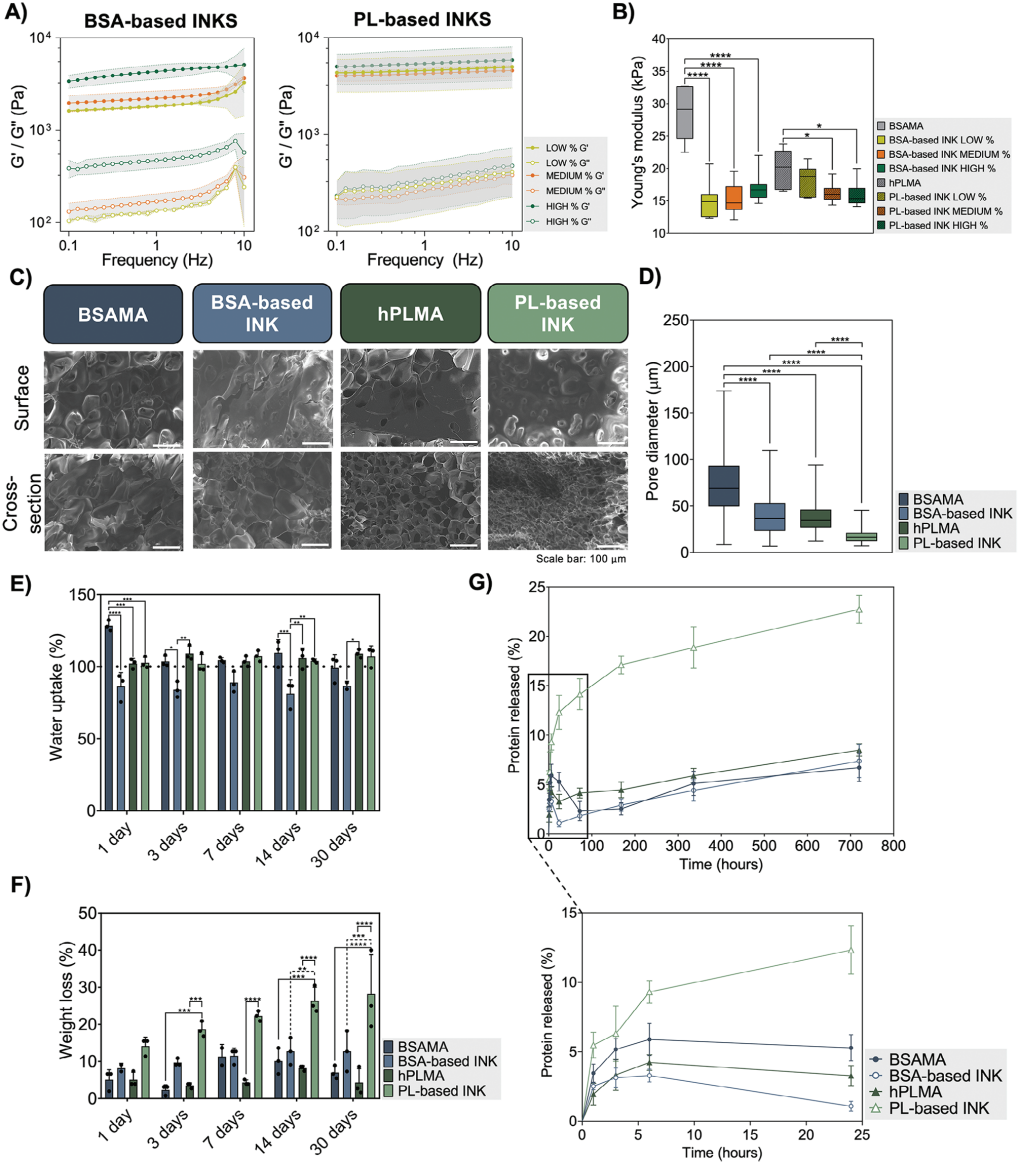
Mechanical and structural properties of photocured structures evaluation. A) Frequency sweeps after photocrosslinking for BSA‐ and PL‐based inks hydrogels. B) Young's modulus calculated after compressive tests of BSAMA 20 wv%, hPLMA 20 wv%, and BSA and PL‐based inks hydrogels (LOW%, MEDIUM% and HIGH%). Statistical analysis through one way ANOVA with multiple comparisons showed significant differences between the conditions under analysis (*n* ≥ 6). C) SEM surface and cross section representative images of freeze‐dried BSAMA 20 wv%, hPLMA 20 wv% and BSA‐ and PL‐based ink MEDIUM% hydrogels. D) Pore size measurements of freeze‐dried structures observed under SEM. Statistical analysis through one way ANOVA with multiple comparisons showed significant differences between the conditions under analysis E) Water uptake (%), F) Weight loss (%), and G) Protein release (%) quantification of BSAMA 20 wv%, hPLMA 20 wv%, and BSA‐ and PL‐based ink MEDIUM% hydrogels. Statistical analysis through 2‐way ANOVA with multiple comparisons showed significant differences between the conditions under analysis (*n* = 3). *p < 0.05, **p < 0.01, ***p < 0.001, and ****p < 0.0001.

After rheological characterization of BSA‐ and PL‐based inks developed in this work, it was also important to evaluate the mechanical properties and structural features of the platforms fabricated upon photocrosslinking. Frequency sweeps after photocrosslinking (**Figure** **3**A) showed an increased moduli when compared with the precursor inks, as well as a moduli dependency on the cross‐linker concentration as corroborated by photorheology (Figure 2J). Increased cross‐linker concentrations display increased moduli values, due to the higher cross‐linking density. For compressive stiffness characterization, photocrosslinked BSA‐ and PL‐based hydrogel inks, as well as photocrosslinked BSAMA 20 wv% and hPLMA 20 wv% hydrogels – here used as controls – were subjected to compressive tests in order to calculate the Young's modulus (Figure 3B). Representative stress/strain curves used for these measurements are depicted in Figure S13. Significant differences were observed between the controls and respective ink hydrogels, with BSAMA and hPLMA hydrogels displaying higher values for the Young's modulus compared with the ink hydrogels. This can be explained by the presence of non‐modified proteins, i.e., BSA and PL, and therefore a decrease in the photocrosslinkable moieties thus leading to a diminished density of covalent bonds.^[^ ^45^ ^]^ Regarding BSA‐ and PL‐based ink hydrogels, although there are no significant differences between all conditions (LOW%, MEDIUM% and HIGH%), it is possible to observe small variations in stiffness depending on the cross‐linker concentration.

In order to assess hydrogels microstructure, often influenced by polymer concentration and cross‐linking mechanisms, SEM analysis of freeze‐dried scaffolds was performed for both BSAMA 20 wv% and hPLMA 20 wv%, as well as scaffolds produced using BSA‐ and PL‐based inks with MEDIUM% cross‐linker concentration, as previously stated by the rheological characterization results as the ideal condition for 3D extrusion printing. Although the freeze‐drying process affects the native internal microstructure of the hydrogels,^[^ ^46^ ^]^ it is possible to observe significant differences regarding pore dimensions of control scaffolds in comparison with ink scaffolds (Figure 3C), with the former showing an internal structure with larger pores than ink conditions (Figure 3D). The presence of a smaller pores network in ink hydrogels is explained by the increased cross‐linking entanglements due to the EDC/NHS cross‐linking that precedes the photocrosslinking process. Moreover, it was possible to observe that PL‐derived hydrogels present a more homogeneous porous network compared to BSA‐based hydrogels. Water uptake behavior of these hydrogels was also explored, showcasing the ability of our hydrogels to retain water over time (Figure 3E). In this case, BSAMA hydrogels displayed increased water uptake compared to all the other conditions under analysis, which we hypothesize being due to the presence of a larger pores network, highly related with increased capacity to absorb and retain water.^[^ ^46^ ^]^

Further characterization regarding the stability of the scaffolds was carried out by studying hydrogel weight loss over time (Figure 3F) as well as protein release quantification (Figure 3G). Our results showed a sustained release of proteins for all conditions, being more accentuated during the first 24 h. PL‐based ink hydrogels showed the highest weight loss, with ≈20% of the total protein content in the hydrogel being released over 30 days. On the other hand, BSAMA, hPLMA and BSA ink hydrogels demonstrate similar protein release profiles, as well as similar weight loss percentages among all conditions. Although PL's most abundant protein is albumin, several smaller proteins are part of their composition,^[^ ^10^ ^]^ which can be more easily released, comparing with BSA conditions, further supporting the highest protein release observed for PL‐based ink hydrogels.

Altogether, these results demonstrate the ability of BSA‐ and PL‐based inks to serve as precursor materials to produce photocrosslinked structures with tunable mechanical properties dependent on the cross‐linker concentrations as well as good in vitro stability and sustained protein release thus being promising platforms for local delivery of proteins to promote cell proliferation and tissue re‐growth.

### 2.4 Filament Fidelity Analysis

**Figure 4:**
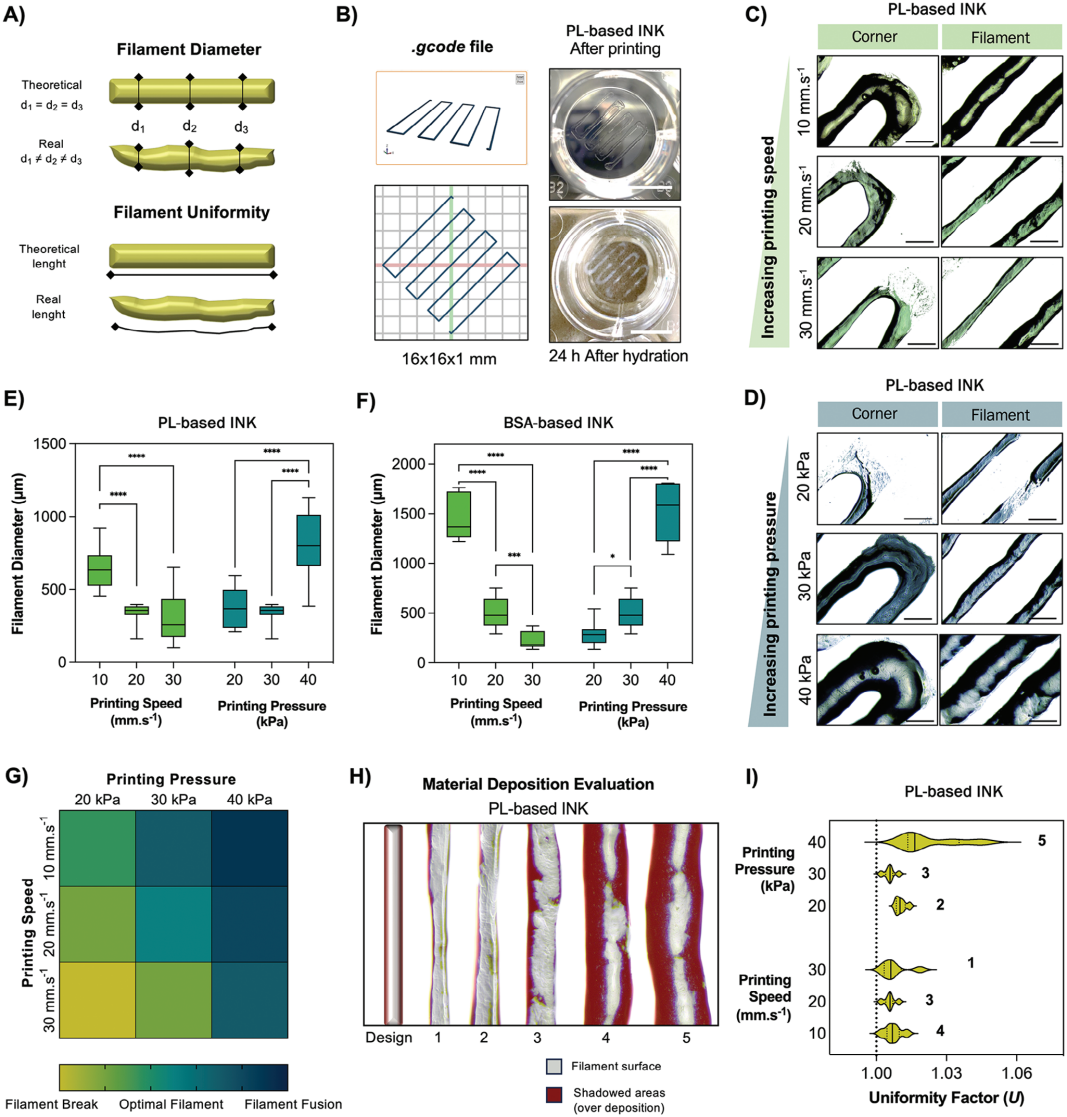
Filament fidelity analysis. A) Schematics of the quantitative tests to assess the filament fidelity of an ink post printing; B) Design of the gcode file used for filament fidelity analysis and visual images of the obtained 1‐layered structures after printing (above) and after 24 h in PBS (below). Scale bars: 1 cm; C,D) representative micrographs of the obtained filaments and corners of PL‐based inks by varying speeds (10, 20, and 30 mm s^−1^) and pressures (20, 30, and 40 kPa), respectively. Scale bars: 1 mm; E,F) Diameter of the filaments obtained using different pressures and speeds on PL and BSA‐based inks, respectively. Filament sizes decrease with the increasing velocity and increase with the increasing pressure. 2way ANOVA with multiple comparisons was used to compare between printing speeds or printing pressures using the same ink (*n* = 10); G) Heatmap obtained by quantification of filament breaks (green) and filament fusion (dark blue) spots. Optimal filament pressures and speeds are indicated in light blue; H) Colorimetric analysis to assess material uniformity deposition. The speeds and pressures used for each filament are depicted in I); I) Filament uniformity quantification (*n* = 6). Numbers are related to the filaments presented in H). ^****^ *p* < 0.001; ^***^ *p* < 0.005; ^*^ *p *< 0.05.

Printability, i.e., the ability of forming filaments, grids and large structures using extrusion‐based 3D printing is one of the most important parameters to evaluate when formulating novel inks. For filament fidelity analysis, we considered extrudability, extrusion uniformity, and also structural integrity of the deposited filaments using the MEDIUM% cross‐linker concentration, as it exhibited the most favorable rheological profile for 3D printing (**Figure 2)**. The performed analysis is schematized in Figure 4A. The size and design of the 1‐layer geometry used for these evaluations is represented in Figure 4B.

Figure 4C,D displays representative replicas of the micrographs used for the measurement of PL‐based filament diameters. The same pictures were taken and used for the measurement of BSA‐based filament diameters (Figure S14, Supporting Information). Overall, results indicate an increase in filament diameter with the decreasing printing velocities and the increasing printing pressure. These results were expected and concordant for both PL‐based inks (Figure 4E) and BSA‐based inks (Figure 4F). Comparing both inks, we could observe that BSA‐based inks had lower filament fidelity when compared to PL inks, i.e., the measured diameter is further from the theoretical value. Additionally, BSA‐based inks were found to be more responsive to the pressure and speed, reaching values of filament diameter ≈1500 µm, which was not ideal. It was interesting to observe that, when compared to the variations in printing velocity, there was a much more pronounced dependency on the pressure for both inks, achieving higher filament diameter values when increasing the applied pressure. Moreover, we detected higher frequency of filament breaks when decreasing the pressure, a result which was further corroborated by heatmap (Figure 4G). This 3D printing pressure dependency was already demonstrated in other reports using natural‐based inks.^[^ ^47^, ^48^, ^49^ ^]^ We have additionally performed filament uniformity analysis by using individual filament micrographs (Figure 4H) to measure real filament length according to Figure 4A. By dividing real by theoretical length of the filament, we obtained values for uniformity ratio (*U*), which are presented in Figure 4I. Although no statistical difference was found between the results, we obtained higher *U* values (i.e., non‐uniform filaments) with the increase of printing pressure (condition n° 5), which was observed not only macroscopically (more sinuous contours), but also on the obtained *U* values and respective dispersion. The remaining values for uniformity were similar between them, although condition n° 3 revealed the least scattered results. We also evaluated the stability of these filaments by immersing the printed layer in distilled water immediately after printing (Figure 4B; Figure S15, Supporting Information). These filaments revealed stability and no significant swelling in aqueous environments after photocrosslinking, further corroborating previous results for scaffold analysis (Figure 3).

It is important to notice that even though the measurement of filament diameters alone did not allow us to verify quantitative differences between the higher printing velocities and pressures, we were able to demonstrate significant discrepancies when accounting filaments merges/breaks and uniformity values. In summary, a multi‐factor analysis was needed for identifying optimal printing pressure and speed values for 3D extrusion printing, as previously reported.^[^ ^50^, ^51^ ^]^ As a result, we established the following optimal printing parameters for further progress: a printing velocity of 20 mm s^−1^ and a printing pressure of 30 kPa.

### 2.5 Shape Fidelity Analysis

**Figure 5:**
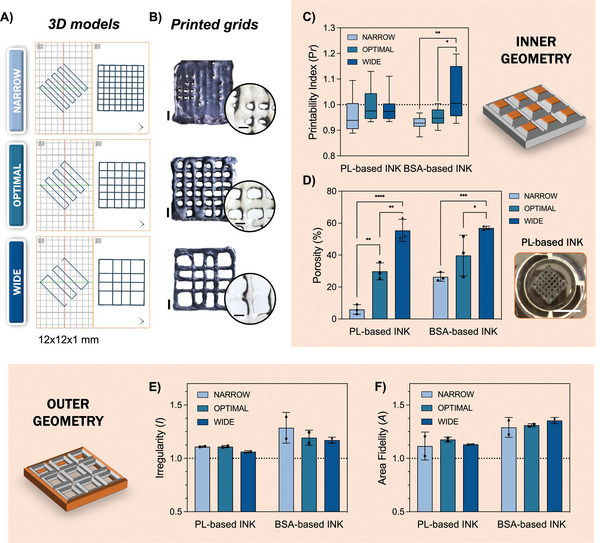
Shape fidelity analysis in grid designs. A) Designs used for 3D printing grids with increasing infill densities (from top to bottom); B) Stereomicroscope images of the obtained structures using PL‐based inks for narrow, optimal, and wide geometries. Scale bar: 2 mm. Zoom‐in image of the pore geometry obtained in each one of the structures. Scale bar: 1 mm; C,D) Evaluation of the inner geometry of the grids by printability index calculations (*n* = 10) and porosity percentage (*n* = 3), respectively, demonstrating printability values closer to 1 and increasing porosity with the lower infill percentage; Small image on the right represents a grid obtained using PL‐based inks using an optimal infill percentage. Scale bar: 1 cm; E,F) Evaluation of the outer geometry of the grids by measuring structures’ area and perimeter irregularity, respectively (*n* = 2). Values demonstrate that infills do not influence outer geometry, but PL‐based inks are closer to ideal fidelity values (1.0). 2way ANOVA with multiple comparisons was used to compare between narrow, optimal, and wide geometries using the same ink. ^****^ *p *< 0.001; ^***^ *p *< 0.005; ^**^ *p *< 0.01; ^*^ *p *< 0.05.

After having the 3D printing parameters set, grid structures of 12 × 12 mm in size were printed for shape fidelity analysis (**Figure** **5**A). These designs allow us to have a clear visualization and measurement of pore geometries (inner geometry) as well as of the overall printing fidelity of the constructs (outer geometry). For the shape fidelity measurements, we studied the influence of 3 infill percentages – here named as Narrow, Optimal and Wide – on obtaining mechanically robust yet porous structures, allowing tissue ingrowth and nutrients exchange. Figure 5B displays a microscopical image of the grids obtained with different infills using the developed PL‐based ink, and a zoom‐in to the obtained pores. Representative images of grids and pores obtained using BSA‐based inks are depicted in Figure S16 (Supporting Information). These images were used to obtain measurements of inner and outer geometries for further printability quantifications.

Analyzing the printability index (*Pr*, Figure 5C), even though no statistical difference was found between the printed designs, there was a marked tendency for the *Pr* to increase inversely to the infill percentage. Additionally, PL‐based inks demonstrated less *Pr* value variability when comparing to BSA‐based inks. *Pr* is a measurement of pore shape fidelity, which is calculated using the perimeter and area of the printed pores. An ideal pore geometry should display a squared profile in the x‐y plane, having a *Pr* value of 1. Thus, values below 1 correspond to round‐shaped pores and values above 1 correspond to irregular pores.^[^ ^51^ ^]^ This clearly corroborates the obtained results, since narrow geometries visually demonstrate merge of the filaments, optimal geometries demonstrate round‐shaped pores (*Pr* < 1) and wide geometries demonstrate irregular‐shaped pores (*Pr* > 1). Still considering inner geometry parameters, a higher value for porosity was found with the decreasing infill percentage, as expected (Figure 5D). Tunable porosities could be valuable for tailoring 3D printed scaffolds to specific applications that need high flow influx, (for instance, highly vascularized tissues), or, on the other hand, that need higher densities of material (for instance for load‐bearing applications or for holding biomolecules that are easily washed out in drug delivery systems).

The outer geometry printability profile was evaluated by performing construct area and irregularity measurements. The area fidelity (A), i.e., the normalization of the measured area value to the area on the initial 3D design, allowed us to conclude that infill percentages do not influence the overall dimensions of the printed structures (Figure 5F). Nevertheless, we were able to observe that BSA printed grids have a pronounced spreading, with area ratio reaching ≈1.3 in value, which was not observed for PL‐based inks (≈1.1 area ratio). Accordingly, the Irregularity (*I*) –, i.e., the ratio between the obtained and the expected square sides – revealed that BSA‐based inks had a noticeable irregular deposition (Figure S16, Supporting Information), which was also not observed for PL‐based inks (*I* < 1.1) (Figure 5E). Moreover, the different infills demonstrated to not significantly influence the outer geometry values in both inks.

Altogether, these results revealed that PL‐based inks demonstrate higher potential as promising ink products from blood components. It is to note that when augmenting the infill, the pores started to close, evidencing that filaments are in general wider than the nozzle size. This could be explained by a three intervals rotational thixotropic test (3ITT, Figure S17, Supporting Information) to determine time‐dependent behaviors on the inks’ viscosity profile, a test that is able to replicate the shear forces at the wall during printing. Figure S17 (Supporting Information) indicates that these inks are not able to recover their initial viscosities upon application of a high stress phase (recovery value ≈30%). This can be translated in a slight thixotropic behavior that is not desirable for extrusion printing, resulting in collapsing of the filaments, lack of shape fidelity and ultimately lack of support for further deposited materials.^[^ ^52^ ^]^ Additionally, the pre‐cross‐linking chemistry applied greatly depends on the number of functional groups available to react, which in turn is dependent on batch‐to‐batch variability. This variability along with the globular nature of these proteins affect the reproducibility and consistency of the pre‐cross‐linking procedure, creating some denser domains of cross‐linking within the ink, sometimes affecting shape fidelity, as seen in Figure 5B. Further improvement of these inks could rely on: 1) applying a faster post‐printing photocrosslinking chemistry, such as photo‐induced thiol‐ene chemistry;^[^ ^53^, ^54^ ^]^ or 2) fostering the creation of dynamic bonds within the ink – instead of static ones – as pre‐cross‐linking strategy. The latter could rely, for instance, in physical linkages such as host‐guest interactions,^[^ ^55^ ^]^ or chemical linkages such as hydrazone bonds,^[^ ^56^ ^]^ among others. Nevertheless, these chemistries require complex synthesis and purification steps, which can compromise the proteins’ bioactivity. Moreover, batch to batch variability will also influence the final functionalization degree, as well as the capacity of having a secondary cross‐linking post‐printing. Overall, taking into consideration the above‐mentioned concerns regarding functionalization scalability, reproducibility, affordability and final bioactivity of the compounds, the herein applied strategy focused on demonstrating a straightforward methodology for the obtention of 3D printable blood‐derived inks that could be easily reproduced.

### 2.6 Printability of Multilayered Geometries

**Figure 6:**
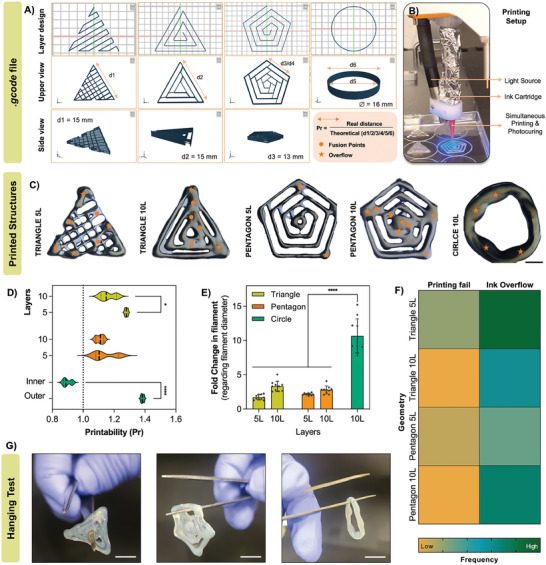
Shape fidelity in multilayered structures. A) Designs used for printing. From left to right: 5 layer (5L) triangle, 10 layer (10L) triangle, 10L pentagon, and 10L circle. d1, d2, d3, d4, d5, and d6 depict the sides that were measured to calculate printability, as indicated in the figure; B) 3D printing setup used in this work; C) Stereomicroscope images of the obtained structures. Filament fusion points and spots of ink overflow are indicated by orange circles and stars, respectively. Scale bar: 0.5 cm; D,E) Evaluation of printing fidelity by calculating printability (*n* = 3) and fold change in filament regarding filament diameter obtained in Figure 4 (*n* = 10); F) Heatmap obtained by quantifying the number of times that filaments were not extruded continuously (printing fails) as well as the number of times that filaments merged, creating over deposition of ink in a certain spot (ink overflow). Frequency of occurrences was colored from orange to green, depicting low to high frequency, respectively; G) Representative images of the hanging test performed after full photocrosslinking of the constructs using forceps. Obtained structures are robust and able to be easily manipulated. Scale bar: 1 cm. 2way ANOVA with multiple comparisons was used to compare differences between number of layers in D) and difference between all printed structures in E). ^****^ *p* < 0.001; ^*^ *p* < 0.05.

Due to the promising results obtained using PL‐based inks, we moved to print several structures with 5 to 10 layers, increasing *z* plane complexity (**Figure** **6**A). The previously optimized parameters for velocity, pressure, and infill were considered on these assays. We were able to obtain triangular, round, and pentagon‐shaped structures (Figure 6C; and Video S1, Supporting Information) with ≈1.5 cm in *x*‐*y* plane with high accuracy, by using a simultaneous printing and photocuring setup (Figure 6B).

After obtaining the constructs, we measured printability by using the formula depicted in Figure 6A. We were able to demonstrate that triangular or pentagon shaped structures were closer to the ideal ratio, when comparing to round‐shaped structures such as the circle, independently of the number of layers (Figure 6D). Additionally, we measured the fold change in filament diameter regarding the results obtained in Figure 4. Once more, we observed that the circle geometry had the highest fold increase (Figure 6E). Additionally, there was a clear tendency (although not statistically significant) for the filament diameter to increase with the increasing number of layers, which may be indicative that the exposure time/intensity of the light was not adequate to fully cross‐link the lower layers before printing the next one. Moreover, other works also reported on coalescence of the printed structures when using natural‐based materials such as alginate and xanthan gum, which can also justify this increase.^[^ ^49^ ^]^ Additionally to the previously reported improvements on the ink, further improvements on the 3D printing process could also be performed. In these printing setups, the printer was not able to retract the filament when changing layers and/or printing spot, which meant that the pressure was still being applied while the nozzle was “on air”. This resulted in an accumulation of material in the spot where the printing begun, giving rise to some inaccuracies on overall printing fidelity. Since the circle is always printing layers above each other, this effect was more pronounced, thereby reflecting not only in the printability values, but also on the filament fold change.

These results were supported by quantifying the number of fusion points (Figure 6C, orange dots), printing overflows (Figure 6C, orange stars) and printing fails, which were further translated in a heatmap (Figure 6F). Results demonstrate overall less frequency of printing fails in regard to overflows. Additionally, ink overflows were more prevalent on the 10‐layered structures, apart from the triangle with 5 layers, in which the inner infill was a more complex grid structure. These results confirm that using these inks, continuous printing favors achieving more consistent and robust constructs, with adequate printing fidelity. Indeed, we were able to easily manipulate printed structures by hand, which did not break or collapse after hanging test (Figure 6G).

We have clearly demonstrated, through 3D printing of filaments, grids and complex constructs that there is certainly the need for evaluating not only the printing speeds and pressures but also the most adequate infill (percentage and geometry) for the desired application and, most importantly, to establish the right printing setup. Two of the factors that have demonstrated very important are the retraction of the filament simultaneous to the change in the printing spot, which avoids ink overflow and printing inaccuracies, and adjusting the light intensity to the optimized printing speed.

### 2.7 In Vitro Cell Culture

**Figure 7:**
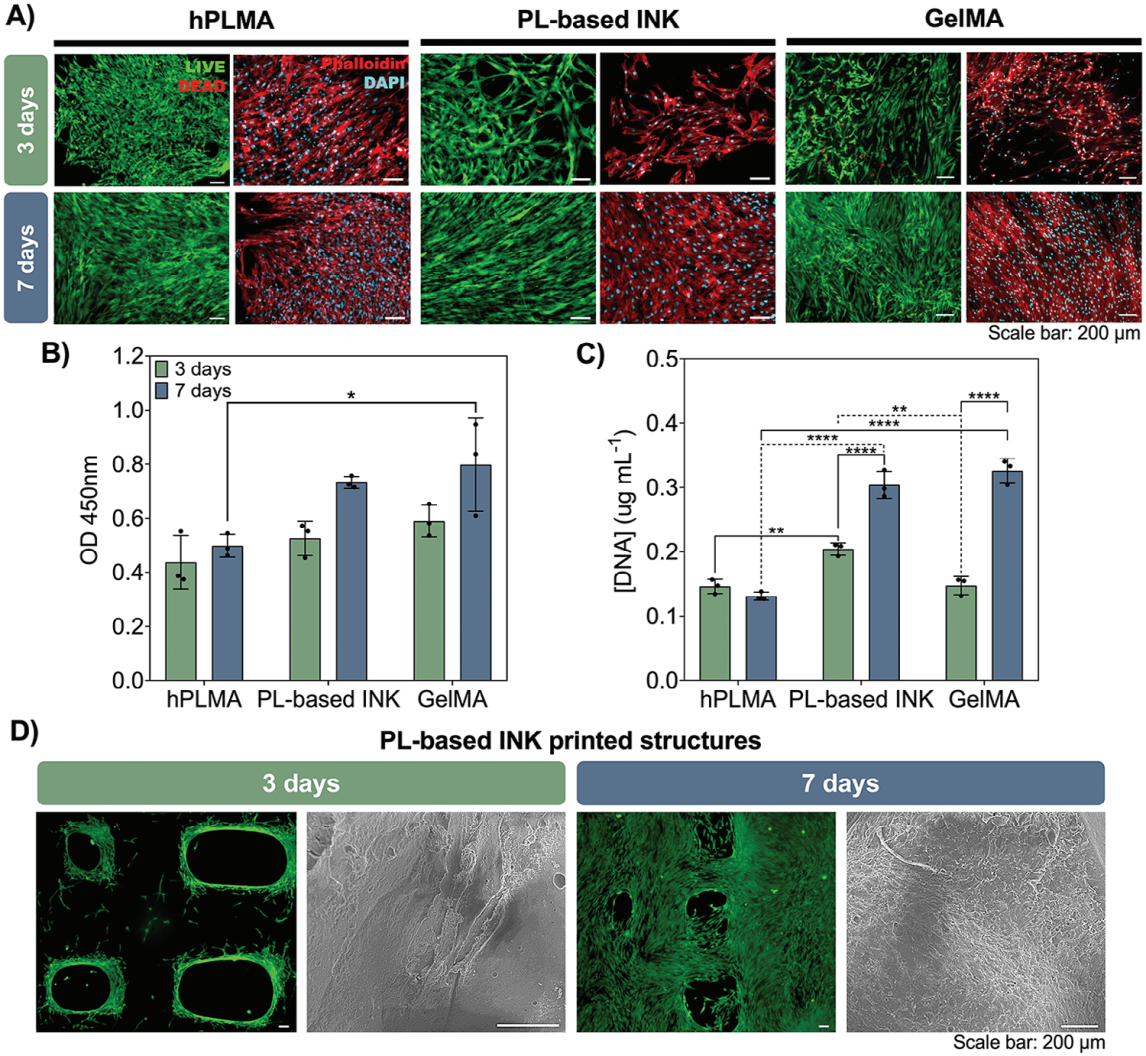
In vitro stem cell culture. A) Representative live/dead and DAPI/Phalloidin staining images of hASCs cultured on top of hPLMA hydrogels, PL‐based inks hydrogels and GelMA hydrogels (positive control) at 3 and 7 days of culture. Scale bar: 200 µm B) CCK‐8 and C) DNA quantification results at 3 and 7 days of hASCs cultured in the hydrogels. Statistical analysis through 2way ANOVA with multiple comparisons showed significant differences between the conditions under analysis (*n* = 3). *p < 0.05, **p < 0.01, and ****p < 0.0001 D) Representative live/dead and SEM images of hASCs seeded on top of PL‐based ink printed constructs after 3 and 7 days of culture. Scale bar: 200 µm.

Human adipose‐derived stem cells (hASCs) were used to assess PL‐based inks cytocompatibility as well as their performance as platforms to support stem cell culture. In this sense, PL‐based inks MEDIUM% were used to produce hydrogels in which hASCs were seeded and cultured for 7 days. Additionally, hPLMA 20 wv% hydrogels were produced and seeded under the same conditions as the ink‐based hydrogels. As control, gelatin methacryloyl (GelMA) hydrogels were fabricated and seeded with hASCs following the same conditions. GelMA is a cost‐effective collagen derivative widely explored for tissue engineering purposes with recognized ability for long term cell culture.^[^ ^57^, ^58^, ^59^ ^]^ As such, GelMA hydrogels were herein produced and used as positive control to better evaluate the cytocompatibility of PL‐based constructs. **Figure** **7**A shows representative images of live/dead assays performed for all culture conditions at 3 and 7 days of culture, showing the ability of hASCs to adhere to the hydrogels and remain viable up to 7 days of culture in all analyzed conditions. Morphological features of the cells cultured on top of the hydrogels were also assessed by staining the F‐actin filaments (phalloidin, red) and nuclei (DAPI, blue) (Figure 7A). hASCs readily elongate after 3 days, and at 7 days are perfectly stretched and able to completely spread across the entire hydrogel surface. When compared to GelMA hydrogels (positive control), PL‐based matrices showed similar behavior regarding cell adhesion and elongation, therefore demonstrating their potential as 3D cell culture platforms. To further confirm cell viability, CCK‐8 test was performed at both time points (Figure 7B). Results showed a slight increase from 3 to 7 days for all conditions under analysis, being possible to confirm that hASCs remain viable up to 7 days when cultured in PL‐based inks hydrogels without significant differences when compared hPLMA hydrogels. Moreover, when compared to the positive control, PL‐based matrices exhibited equivalent performance thus validating their cytocompatibility and potential use for cell culture and tissue engineering purposes. DNA quantification (Figure 7C) results showed similar tendency with a slight increase in DNA content throughout cell culture time, thus corroborating the above disclosed results of cell viability assays.

After validating the developed inks for generating hydrogels capable of supporting stem cell culture, it is important to explore their use to fabricate 3D printed structures that can ensure cell maintenance and support proliferation. To do so, PL‐based inks MEDIUM% were prepared and subjected to extrusion 3D printing. Following photocrosslinking of the final constructs, hASCs were seeded and live/dead was performed at pre‐determined time‐points. In Figure 7D representative live/dead images show hASCs adhered to PL‐based ink printed structures after 3 days of culture, these being able to proliferate over time and cover the entire surface of the construct after 7 days in culture, as indicated also by SEM micrographs. Additionally, tests were performed with PL‐based inks laden with hASCs (Figure S18, Supporting Information). Although some cell survival can be observed 24 h after printing, some cell death can also be seen (red staining), showing the poor ability of PL‐based inks to be used as bioinks. Besides the fact that the range of shear forces used for printing can affect cell survival,^[^ ^60^ ^]^ the unreacted EDC or reaction by‐products that are not fully released from the bioink are also possible explanations for this phenomenon.^[^ ^61^ ^]^

PL‐based inks herein developed demonstrate great potential as precursor materials for extrusion 3D printing, being possible to generate matrices with proved cytocompatibility. Although some limitations can be pointed out regarding the use of PL‐based inks, our preliminary data on the use of these inks to fabricate human‐derived constructs for stem cell culture reveals the ability of such platforms for cell maintenance, thus offering a new possibility to use biocompatible human blood‐derived materials for the development of customized tissue engineering systems using 3D printing.

## 3 Conclusion

We herein introduced the use of blood components as a biomaterial for 3D extrusion (bio)printing strategies for the first time. The synergistic combination of photocurable with non‐photocurable matrices enabled the development of programmable viscoelasticity and shear‐thinning properties on pre‐gels by leveraging zero‐length cross‐linkers. This unique material combination also allowed post‐printing photocuring and hardening of the structure without using toxic substances and/or plastic materials. We have additionally noticed that platelet lysate‐based inks exhibit higher reproducibility when comparing with the albumin‐based ink products. The herein presented ink engineering strategy denotes an easy‐to‐handle, tunable, biocompatible and sustainable solution for 3D printing natural‐based materials that can be applied on every extrusion printer. Indeed, because these components can be obtained from any patient, it unlocks the possibility to create patient‐specific treatments that match the regenerative requirements of native tissues. These human derived proteins can additionally be obtainable from renewable sources using environment‐friendly manufacturing processes, ensuring reduced carbon footprint and economical benefit to society.

## 4 Experimental Section

Additional materials & methods are available in Supporting Information.

### PL and BSA‐Based Ink Manufacturing Process

Inks were produced using a total protein concentration of 10 wt.% for BSA‐based inks and 20 wv% for PL‐based inks. The rationale for ink engineering requires chemical coordination between the photopolymerizable network and the non‐modified network of the same protein (Figure 1). So briefly, BSA or PL were dissolved in PBS at the above‐mentioned concentrations together with LAP at 1 wt.% for 30 min at RT. Separately, BSAMA and hPLMA were also dissolved using the same volume as their non‐modified counterparts to further mix them at a 1:1 volume ratio. Right before mixing, EDC and NHS are also dissolved in PBS at 100 and 25 wt.%, respectively, so that the final mass ratio of these reagents in the ink is 1:0.25. This was performed in order to neglect difference in volumes to be added to the final ink, which could be affecting chemical coordination. With all reagents set, EDC and NHS were added sequentially to BSAMA or hPLMA at predefined concentrations by a preliminary screening, according to the protein's degree of methacrylation. In this case, they were added at a 1.3–1.5 vv% and 2.2–2.6 vv% for BSA and PL‐based inks, respectively, and mixtures were toughly vortexed in‐between and in the end. This mixture was left to react for 5 min in a Thermomixer (Eppendorf, VWR) at 1000 rpm, creating an amine‐reactive mixture. After these 5 min, BSA or PL were added to the mixture at a 1:1 volume ratio, so that the final concentration of LAP is 0.5 wt.%. The final BSA and PL‐based inks were obtained by continuous agitation in a Thermomixer, maintained at 37 °C and 1000 rpm overnight.

### Rheological Characterization

Evaluation of the inks rheological proprieties was performed on 2.0, 2.2, and 2.4 vv% EDC/NHS for PL‐based inks and 1.2, 1.3, and 1.4 vv% of EDC/NHS for BSA‐based inks. The assays were performed using a rheometer (Kinexus Prot, Malvern). Twenty millimeters diameter parallel plates and 0.5 mm gap size was applied for shear rate and shear strain sweeps. Eight millimeters diameter parallel plates and 1 mm gap size was applied for the remaining measurements. Strain sweeps from 0.01% to 100% were performed at 1 Hz to determine the linear viscoelastic region (LVER), and a strain of 0.1% was set for the remaining sweeps. Shear rate sweeps were conducted to assess viscosity, by varying the shear rate from 0.01 to 1000 s^−1^. Photorheology analysis was performed on the inks by time sweep – setting the strain to 0.1% and frequency to 1 Hz – using a UV curing system attached to the rheometer and a glass bottom plate (OmniCure S2000, Excelitas Technologies Corp., USA, wavelenght 300–500 nm, 1000 mW cm^−2^ intensity). Dynamic frequency sweep analysis was performed to the inks and also to the scaffolds, in this case by applying 180 s of light exposure (at the same conditions as described above). Frequency sweep was performed from 0.01 to 100 Hz. Rotational three interval thixotropic test (3ITT) was performed to MEDIUM% PL‐based inks in an 8 mm diameter plate with 1 mm gap size. A fixed shear rate of 0.1 s^−1^ was applied to the first and last intervals whereas a fixed shear rate of 1010 s^−1^ was applied to the second interval. Recovery percentage was calculated dividing final by initial plateau viscosity values as indicated in Figure S17 (Supporting Information). All assays were performed at room temperature with three independent experiments and using mineral oil (Fisher Scientific) as solvent trap in the bottom plate. Origin software (2022) was used to retrieve flow behavior and consistency index by non‐linear fitting of viscometry shear rate sweeps to the power law model (Allometric1 function). Yield stress was obtained by non‐linear fitting shear stress shear rate sweeps to the Hershel‐Buckley model (Rheology functions). Tan delta was calculated by dividing G’’/G’ values at 1% shear strain (with exception of LOW%, in which the values were retrieved at 0.1% strain, due to the loss of the linear viscoelastic properties at higher strains). Shear rate at the wall was calculated according to previously described equations.^[^ ^42^, ^43^ ^]^

### Extrudability

Extrudability tests were performed using a Syringe pump (Harvard Apparatus), by setting the flow rate at 0.5 mL min^−1^. Conical nozzles (22, 25, and 27G) as well as 25G metallic nozzle were used to access the most suitable printing tip. Shear rate at the wall was calculated as described elsewhere.^[^ ^42^, ^43^, ^50^ ^]^

### 3D Printing Setup and Printing Fidelity Analysis

For 3D printing, a GeSiM BioScaffold Printer 3.3 Prime was used. Briefly, inks were removed from the thermomixer, placed in 3 mL cartridges and centrifuged for 5 min at 1500 rpm. 25G conical gauges were used, according to the results obtained for extrudability. Printing models are depicted in Figures 4, 5, 6. A velocity of 20 mm s^−1^ and a pressure of 30 kPa were set as printing conditions after filament fidelity analysis. Simultaneous printing and photocuring was performed (setup presented in Figure 6), using a photocuring source (365 nm) at an intensity of 46 mW cm^−2^, and at a medium distance of 2 cm from the printing spot. Equations used for printing fidelity analysis are presented in Supporting Information.

### Mechanical Analysis

The mechanical behavior of PL and BSA‐based inks (same conditions as rheological evaluation) was assessed by compression testing using a Instron Uniaxial Testing Machine (INSTRON, US) equipped with a 50 N load cell. Unidirectional compression assays were performed after 40 s photocrosslinking (VALO LED Curing light, wavelength 400–500 nm, 1000 mW cm^−2^ intensity) in freshly prepared cylindrical hydrogels (6 mm of diameter and 2 mm of height). The Young's modulus was defined as the slope of the linear region (0–5% of strain) of the strain‐stress curve.

### Water Uptake and Weight Loss Analysis

Water uptake, weight loss and protein release of photocrosslinked PL and BSA‐based inks and respective controls (hPLMA and BSAMA photocrosslinked hydrogels) (*n* = 5 for each condition) was performed in sterile conditions by immersing hydrogels in PBS and incubating them at 37 °C. At timepoints 1‐, 7‐, 14‐, 30‐days hydrogels were taken from PBS and the samples were weighted in their wet state (*M_w_*). The PBS was also collected and stored at −20 °C for further protein release quantification analysis. Afterward, samples were then frozen and lyophilized to obtain their dry weight (*M_d_*). Water uptake and weight loss were calculated as follows:

(1) $$Water\;uptake\;\left(\%\right)\;=\frac{M_{w}\;\left(t_{0}\right)-M_{w}\;\left(t_{1}\right)}{M_{w}\;\left(t_{0}\right)}\;\times 100$$

(2) $$Weight\;loss\;\left(\%\right)\;=\frac{M_{d}\;\left(t_{0}\right)-M_{d}\;\left(t_{1}\right)}{M_{d}\;\left(t_{0}\right)}\;\times 100$$

Total protein quantification was performed to the previously frozen PBS that was in contact with samples by using Micro BCA Protein Assay Kit (Thermo Fisher Scientific, USA). Protein release (%) was calculated by comparison with the initial mass of protein in the hydrogel.

### Structural and Morphological Analysis

Structural properties of hydrogels of PL and BSA‐based inks and respective controls (hPLMA and BSAMA) were assessed by scanning electron microscopy (SEM). Cylindrical hydrogels (6 mm diameter, 2 mm height) were produced after 40 s photocrosslinking and frozen at −80 °C. Samples were then freeze‐dried and imaged via SEM using a Hitachi SU‐3800 (Hitachi, Japan). SEM images were analyzed by ImageJ software for pore size measurements.

### In Vitro hASCs Culture

For cell culture assays, cylindrical hydrogels (6 mm diameter, 2 mm height) of hPLMA PL‐based inks were fabricated. For this purpose, hPLMA solution at 20 wv% in 0.5 wv% LAP in PBS was made and hydrogels were fabricated by photocrosslinking. PL‐based inks MEDIUM% were produced as described in *PL and BSA‐based ink manufacturing process* section, and the final hydrogels were produced by photocrosslinking. As control, gelatin methacryloyl (GelMA) hydrogels were produced by photocrosslinking of 10 wv% GelMA solution. Once the hydrogels were fabricated, they were incubated overnight in α‐MEM supplemented with 10 vv% FBS and 1 vv% antibiotic/antimycotic. Afterward, hASCs were seeded on top of the hydrogels at a density of 5 × 10^5^ cells cm^−2^. To each hydrogel, 20 µL of cell suspension were carefully placed on top of the scaffold and after 4 h, α‐MEM supplemented with 10 vv% FBS and 1 vv% antibiotic/antimycotic was added. Cells were cultured for 7 days in a humidified 5% CO_2_ atmosphere incubator at 37 °C and the medium was changed every 2–3 days. For 3D printed constructs, PL‐based inks MEDIUM% was placed in a 3 mL cartridge and printed using BIO X 3D printer (Cellink). Grids (10 × 10 mm) were printed with a 25G conical gauge and after photocrosslinking incubated overnight in α‐MEM supplemented with 10 vv% FBS and 1 vv% antibiotic/antimycotic. Afterward, hASCs were seeded at a density of 2.5 × 10^5^ cells cm^−2^ and cultured for 7 days in a humidified 5% CO_2_ atmosphere incubator at 37 °C and the medium was changed every 2–3 days. PL‐based inks MEDIUM% were afterward used in a bioink approach. hASCs were resuspended in the ink at a density of 10 × 10^6^ cells mL^−1^ and the final bioink was placed in a 3 mL cartridge and printed using BIO X 3D printer (Cellink).

### Live/Dead Assay

At pre‐determined time‐points (3 and 7 days of culture), Live/Dead assay was performed. Hydrogels were incubated in a solution of 2 µL of Calcein AM (4 mM in DMSO) and 1 µL of PI (1 mg mL^−1^) in 1 mL of PBS during 30 min at 37 °C. After washing with PBS, hydrogels were examined using a fluorescence microscope (Leica DMi8, Leica Microsystems, Germany). Image processing was performed using LAS X Office software (Leica Microsystems).

### Cell Morphology Assessment

DAPI/Phalloidin staining was performed to assess cell morphology. At pre‐determined time‐points hydrogels were washed with PBS and fixed with a 4 vv% PFA solution in PBS. A phalloidin solution 1:40 in PBS was prepared and the hydrogels were incubated at room temperature for 30 min. After washing with PBS, a DAPI solution was diluted 1:1000 in PBS and the hydrogels incubated during 5 min at room temperature. Afterward, hydrogels were examined using a fluorescence microscope (Fluorescence Microscope Zeiss, Axio Imager 2, Zeiss, Germany). Image processing was performed using ZEN v3.1 blue edition software (Carl Zeiss Microscopy GmbH).

### Cell Viability Assessment

CCK‐8 was here used to evaluate cell viability. At pre‐determined time‐points, the hydrogels were incubated in a solution of CCK‐8 reagent diluted in medium following manufacturer instructions. After 4 h incubation at 37 °C, absorbance at 450 nm was measured using a microplate reader (SpectraMax iD3 Multi‐Mode, Molecular Devices, USA).

### Cell Proliferation Assessment

After cell lysis, total DNA quantification was performed using a Quant‐iT PicoGreen dsDNA kit. At pre‐determined time‐points, hydrogels were washed with PBS, incubated in sterile deionized water and frozen at −80 °C. To induce cell disruption, freeze (‐80 °C) and thaw (37 °C) cycles were performed. Samples were processed according to the specifications of the kit and DNA standards were prepared with concentrations between 0 and 2 µg mL^−1^ from the dsDNA solution provided in the kit. After 10 min incubation in the dark at room temperature, fluorescence was measured at an excitation wavelength of 480 nm and an emission wavelength of 528 nm (SpectraMax iD3 Multi‐Mode).

### Statistical Analysis

Statistical analysis were performed using GraphPad Prism 9 (GraphPad Software, California, USA). Data were expressed as means ± standard deviation of experiments with at least three independent assays. Differences between groups were analyzed by unpaired t test or two‐way analysis of variance in case of experiments conducted over time, using Tukey test for post hoc assessments of differences between samples. Statistical significance was defined as *p* < 0.05.

## Conflict of Interest

The authors declare no conflict of interest.

## Supporting information



Supporting Information

Supplemental Video 1

## Data Availability

The data that support the findings of this study are available from the corresponding author upon reasonable request.
