# Supplementary material for: Leveraging Blood Components for 3D Printing Applications Through Programmable Ink Engineering Approaches
Source: Adv Sci (Weinh). 2024 Oct 25;11(47):2406569. doi: 10.1002/advs.202406569 (PMC11653660; doi:10.1002/advs.202406569)
Supplement: Supplementary file 1 — Supporting Information [file ADVS-11-2406569-s001.pdf]

ADVANCED SCIENCE
Open Access

Supporting Information

for *Adv. Sci.*, DOI 10.1002/advs.202406569

Leveraging Blood Components for 3D Printing Applications Through Programmable Ink Engineering Approaches

Rita Sobreiro-Almeida, Sara C. Santos, Monize C. Decarli, Marcelo Costa, Tiago R. Correia, Joanna Babilotte, Catarina A. Custódio, Lorenzo Moroni and João F. Mano**

Supplementary Materials & Methods

Materials: Methacrylic anhydride (MA), bovine serum albumin (BSA), phosphate buffered saline (PBS, tablets), sodium carbonate anhydrous (Na_2CO_3), sodium bicarbonate ($NaHCO_3$), gelatin methacryloyl (GelMA, 300 g Bloom, 80% degree of substitution) and paraformaldehyde (PFA) were purchased from Sigma Aldrich. Lithium Phenyl(2,4,6-trimethylbenzoyl)phosphinate (LAP), 1-(3-Dimethylaminopropyl)-3-ethylcarbodiimide hydrochloride (EDC) and N-Hydroxysuccinimide (NHS) were purchased from TCI Chemicals. Sodium Hydroxide (tablets, NaOH) was purchased from LabChem. Human Platelet Lysates (PL) were acquired from STEMCELL Technologies. Human methacryloyl platelet lysates (hPLMA) were provided by Metatissue, Portugal in the scope of H2020 InterLynk grant agreement (see acknowledgements section). SnakeSkin dialysis tubes (MWCO 3.5 kDa), Fluoraldehyde™ o-Phthaldialdehyde Reagent Solution (OPA) were obtained from Fisher Scientific. 0.22 mm PTFE filters (Sartolab® P20) were obtained from Sartorius. Antibiotic/antimycotic, Minimum Essential Alpha Medium (α-MEM), Fetal Bovine Serum (FBS), Propidium iodide (PI), 4',6-diamidino-2-phenylindole dihydrochloride (DAPI) and Quant-iT PicoGreen dsDNA kit were purchased from Thermo Fisher Scientific. Collagenase type I was purchased from MP Biomedicals. Phalloidin (Flash Phalloidin™ Red 594, 300U) was purchased from Biolegend. Cell Counting Kit-8 (CCK-8) was obtained from MedChemExpress.

Synthesis of bovine serum albumin methacrylate (BSAMA): BSA methacrylation was carried out according to previously published protocols.[1] Briefly, BSA was dissolved in carbonate bicarbonate buffer at 5 wt% at 37 °C for 1h. After this, pH was adjusted to 9 with 5M NaOH. While stirring, add MA in a dropwise manner at a 10 vv% to BSA. The solution was left to react at 500 rpm, 37 °C during 1h, after which pH was adjusted to 7.4. This solution was dialyzed against distilled water using a 3.5 KDa membrane for 72h. The purified solution was filtered using 0.22 mm PTFE filters, frozen at -80 °C and freeze-dried for at least 7 days. The obtained powder was stored at 4 °C and sealed with parafilm to prevent moisture.

PL powder preparation: Before use, PLs were diluted in water at a 33 vv% and aliquoted in 50 mL falcon tubes with filter cap to maintain sterility. After that, they were frozen at -80 °C and freeze-dried for 7 days. The obtained powder was stored in the same conditions as BSAMA for not more than 6 months.

^1H Nuclear Magnetic Ressonance (NMR): NMR analysis was used to verify the effective functionalization of original PL and BSA proteins. Solutions of 10 mg mL^{-1} of non-modified PL and BSA and of hPLMA and BSAMA in methyl sulfoxide-*d6* (DMSO) 99.5% and deuterium oxide (D_2O) (Fisher Scientific, USA) were prepared, respectively, and analyzed by ^1H-NMR with 18 s relaxation delay and 300 scans. The ^1H NMR spectra were recorded on a Bruker AMX 300 spectrometer at 300.13 MHz and analyzed using MestreReNova Software.

Degree of methacrylation with OPA colorimetric assay: Fluoraldehyde™ o-Phthaldialdehyde Reagent Solution was used to assess both BSAMA and hPLMA degree of methacrylation according to manufacturer's instructions. Briefly, 500 µg mL^{-1} solutions of BSA, BSAMA, PL and hPLMA in PBS were prepared. Afterwards OPA reagent was added in a 1:10 (sample:reagent) ratio and the samples were incubated for 5 minutes at room temperature. Fluorescence was measured at excitation 330-390nm and emission

at 436-475 using a microplate reader (SpectraMax iD3 Multi-Mode, Molecular devices, USA). BSAMA and hPLMA modification degree was calculated as follows:

$$Modification\ degree\ (\%) = \frac{PL - hPLMA}{PL} \times 100$$

Printing fidelity evaluation: The following equations were used to measure printability of the obtained structures. Schemes of the performed measurements and calculations are provided in Figures 4, 5 and 6. Porosity was calculated using ImageJ software with color threshold analysis.

$$Uniformity\ factor\ (U, filaments) = \frac{|Experimental\ lenght\ of\ printed\ strand_Y|}{Theroretical\ lenght\ of\ the\ strand}$$

$$Printability\ Index\ (Pr, grids) = \frac{(perimeter\ of\ the\ pore)^2}{16\ \times\ area\ of\ the\ pore}$$

$$Irregularity\ (I, grids) = \frac{|Experimental\ lenght\ of\ printed\ perimeter|}{Theroretical\ lenght\ of\ the\ perimeter}$$

$$Area\ fidelity\ (A, grids) = \frac{|Experimental\ area\ of\ printed\ construct|}{Theroretical\ area\ of\ the\ construct}$$

$$Printability\ (Pr, large\ constructs) = \frac{|Experimental\ lenght|}{Theroretical\ lenght\ (d1/d2/d3/d4/d5/d6)}$$

Stem cell isolation from adipose tissue: Human adipose-derived stem cells (hASCs) were isolated from human adipose tissue following previously reported protocols.[2,3] Liposuction tissue was obtained under a cooperation agreement between University of Aveiro and Hospital da Luz (Aveiro, Portugal), approved by the Ethics Commission and an informed consent was obtained from the donor. The sample was transported in PBS supplemented with 1 vv% antibiotic/antimycotic and stored at +4 °C until isolation. For hASCs isolation, the lipoaspirate sample was washed with PBS and afterwards incubated for 45 minutes with a 0.1 wv% collagenase type I solution in a shaking water bath at 37 °C. After incubation, the solution was centrifuged at 1200 rpm for 10 min. The supernatant was discarded, and the pellet was resuspended in PBS and centrifuged at 1200 rpm for 10 min. The supernatant was then removed and the pellet containing the isolated hASCs was resuspended in α-MEM supplemented with 10 vv% FBS and 1 vv% antibiotic/antimycotic and transferred to a cell culture flask. Cells were cultured in a humidified 5% CO_2 atmosphere incubator at 37ºC. Culture medium was changed after 24 hours and then every 2-3 days.

Supplementary Figures

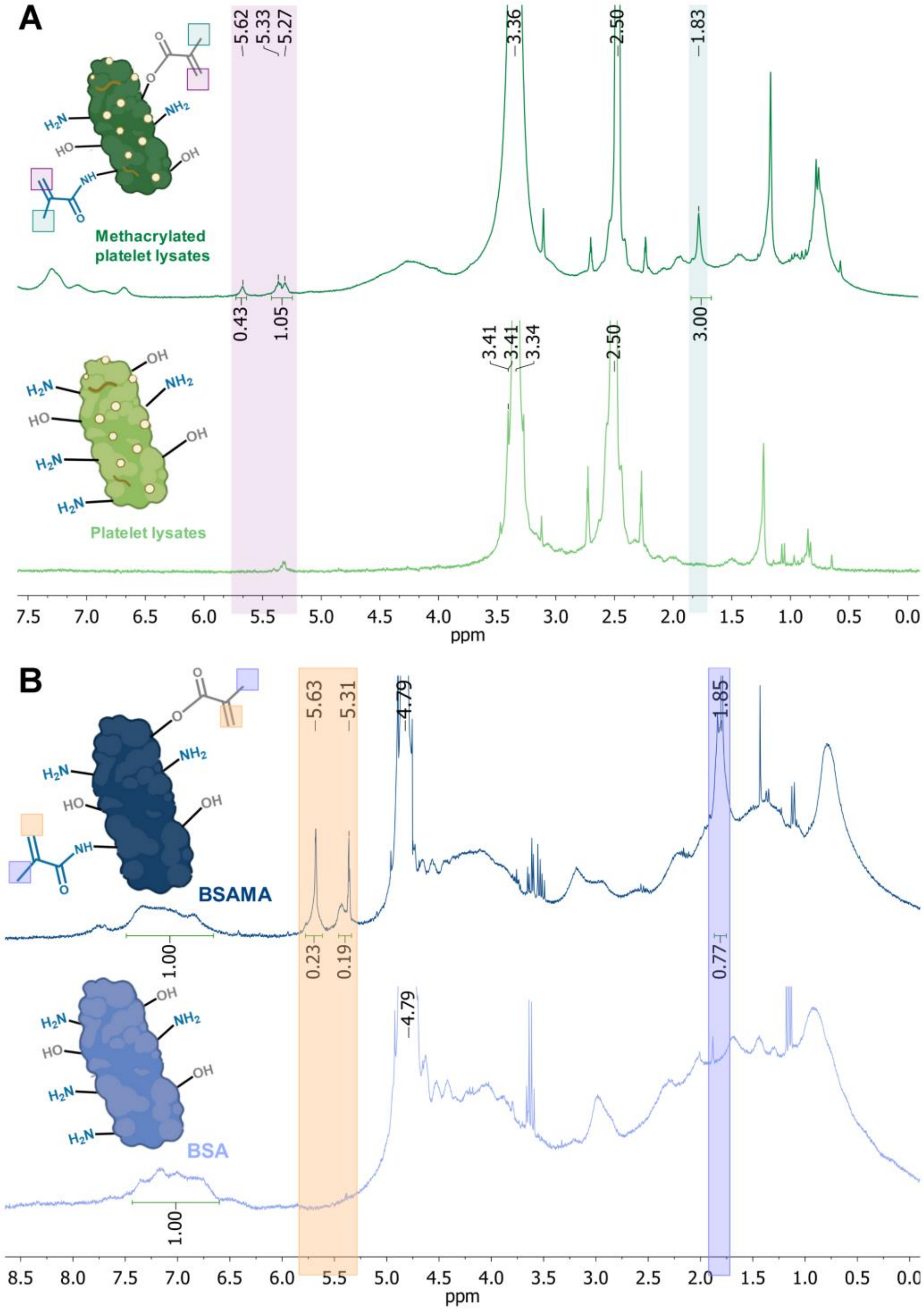

Figure S1: NMR spectra of protein-based materials: (A) non-modified PL and hPLMA; and (B) non-modified BSA and BSAMA.

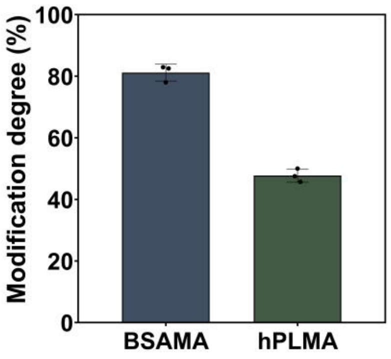

Figure S2: Degree of modification obtained by OPA colorimetric assays (n=3).

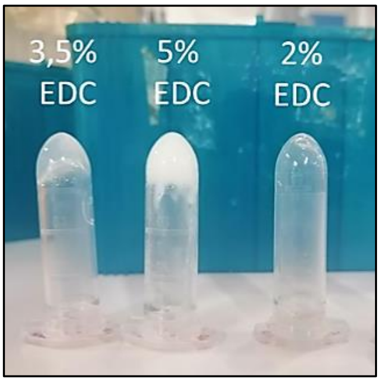

Figure S3: Related to Table S2, this image is representative of the gels that were formed after 5h in MES. A white-ish color, as indicated in this figure (right- and middle-placed eppendorfs) indicated protein precipitation/agglomeration and therefore were not useful as 3D printing formulations.

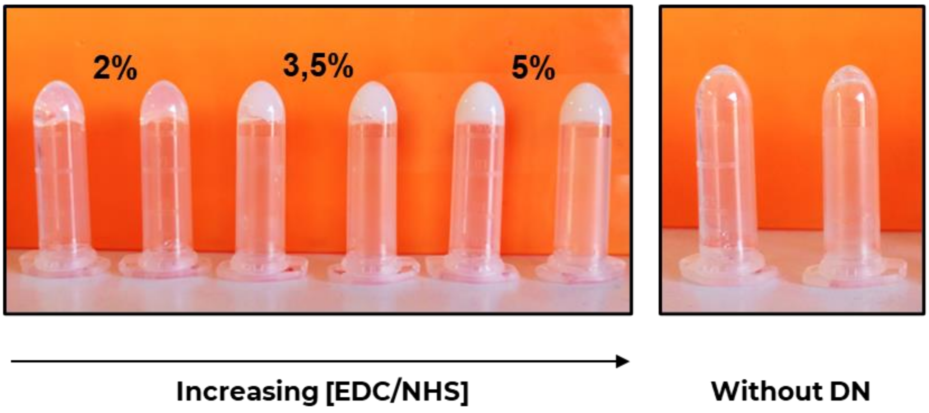

Figure S4: Macroscopical demonstration of the conditions tested in Table S3 with and without using double networks and with different EDC/NHS concentrations.

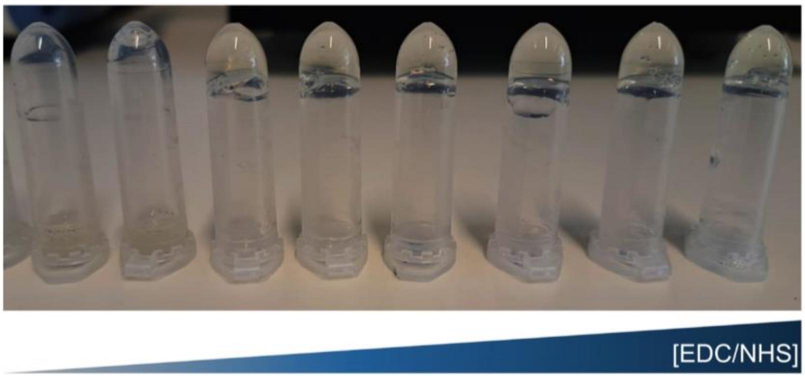

Figure S5: Screening of crosslinker concentrations for printable BSA-based inks by vial inversion test.

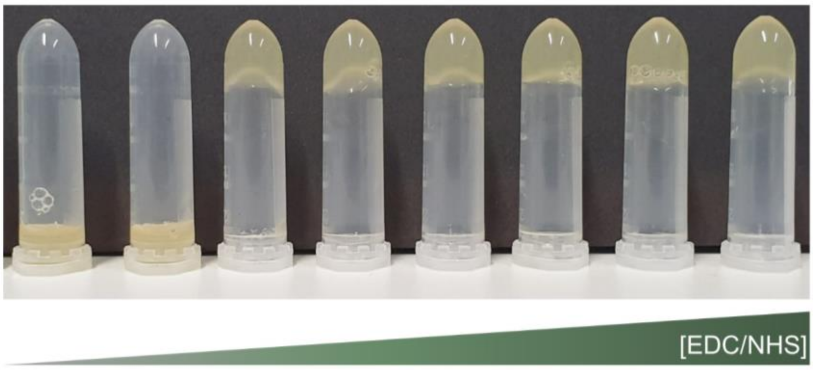

Figure S6: Screening of crosslinker concentrations for printable PL-based inks by vial inversion test.

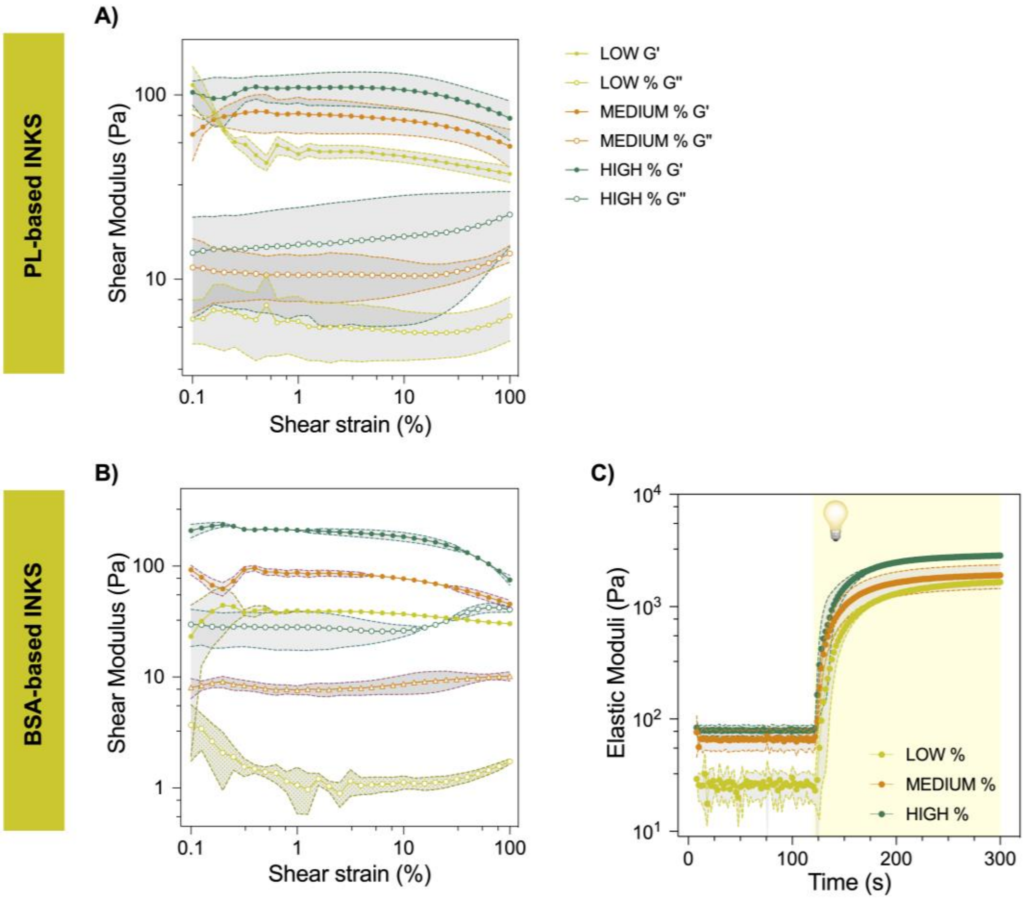

Figure S7: Amplitude sweeps of PL (A) and BSA-based (B) inks for determining the linear viscoelastic region (LVER) (n=3). C) Photorheology of BSA-based inks. Time sweep performed at 0.1% strain and 1 Hz frequency with light irradiation at t=120 s (n=3).

A)

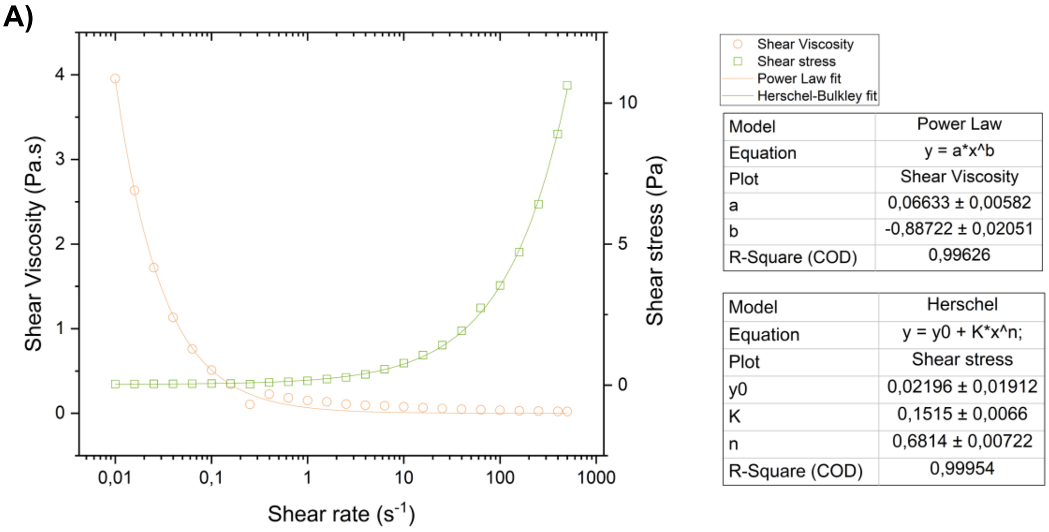

Model	Power Law
Equation	y = a*x^b
Plot	Shear Viscosity
a	0,06633 ± 0,00582
b	-0,88722 ± 0,02051
R-Square (COD)	0,99626

Model	Herschel
Equation	y = y0 + K*x^n;
Plot	Shear stress
y0	0,02196 ± 0,01912
K	0,1515 ± 0,0066
n	0,6814 ± 0,00722
R-Square (COD)	0,99954

B)

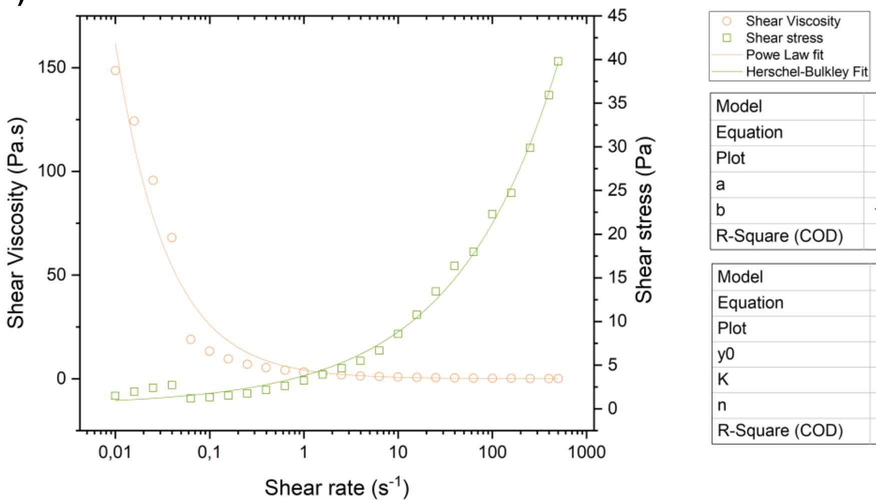

Model	Power Law
Equation	y = a*x^b
Plot	Shear Viscosity
a	4,23367 ± 1,02277
b	-0,79119 ± 0,05723
R-Square (COD)	0,96506

Model	Herschel
Equation	y = y0 + K*x^n;
Plot	Shear stress
y0	0,38531 ± 0,3503
K	3,40627 ± 0,29962
n	0,39338 ± 0,01414
R-Square (COD)	0,99582

C)

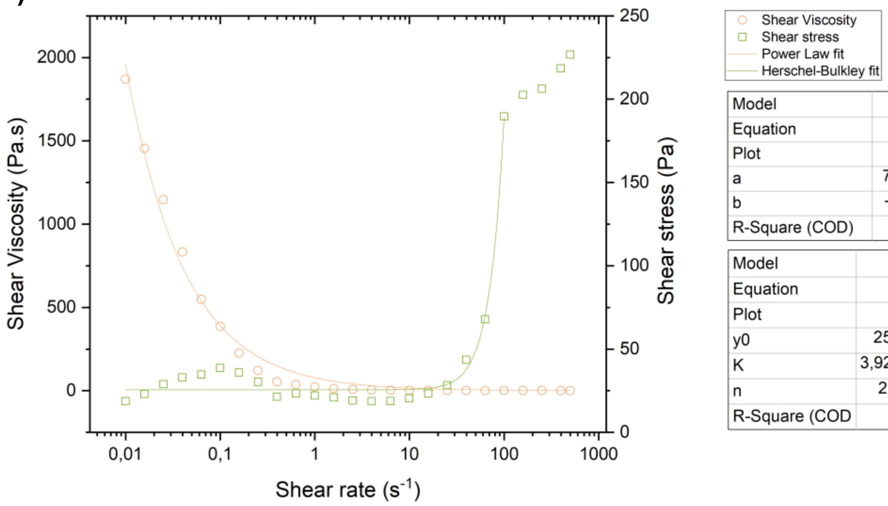

Model	Power Law
Equation	y = a*x^b
Plot	Shear Viscosity
a	77,75299 ± 8,47515
b	-0,70077 ± 0,02622
R-Square (COD)	0,99006

Model	Herschel
Equation	y = y0 + K*x^n;
Plot	Shear stress
y0	25,47438 ± 1,56624
K	3,92596E-4 ± 5,31572E
n	2,81021 ± 0,29549
R-Square (COD	0,97178

Figure S8: Fitting curves for A) LOW%, B) MEDIUM% and C) HIGH% BSA-based inks. All models were fit to the whole range of the curves, with good fitting values (R-square > 0.97) and consistent model predictions throughout (orange lines: power law fit; green lines: Herschel-Bulkley fit), except for the Herschel-Bulkley model on HIGH% inks, that

failed to predict the behavior at high shear rates (fitting range 0.01-100 s^{-1}) (n=3, mean values are represented).

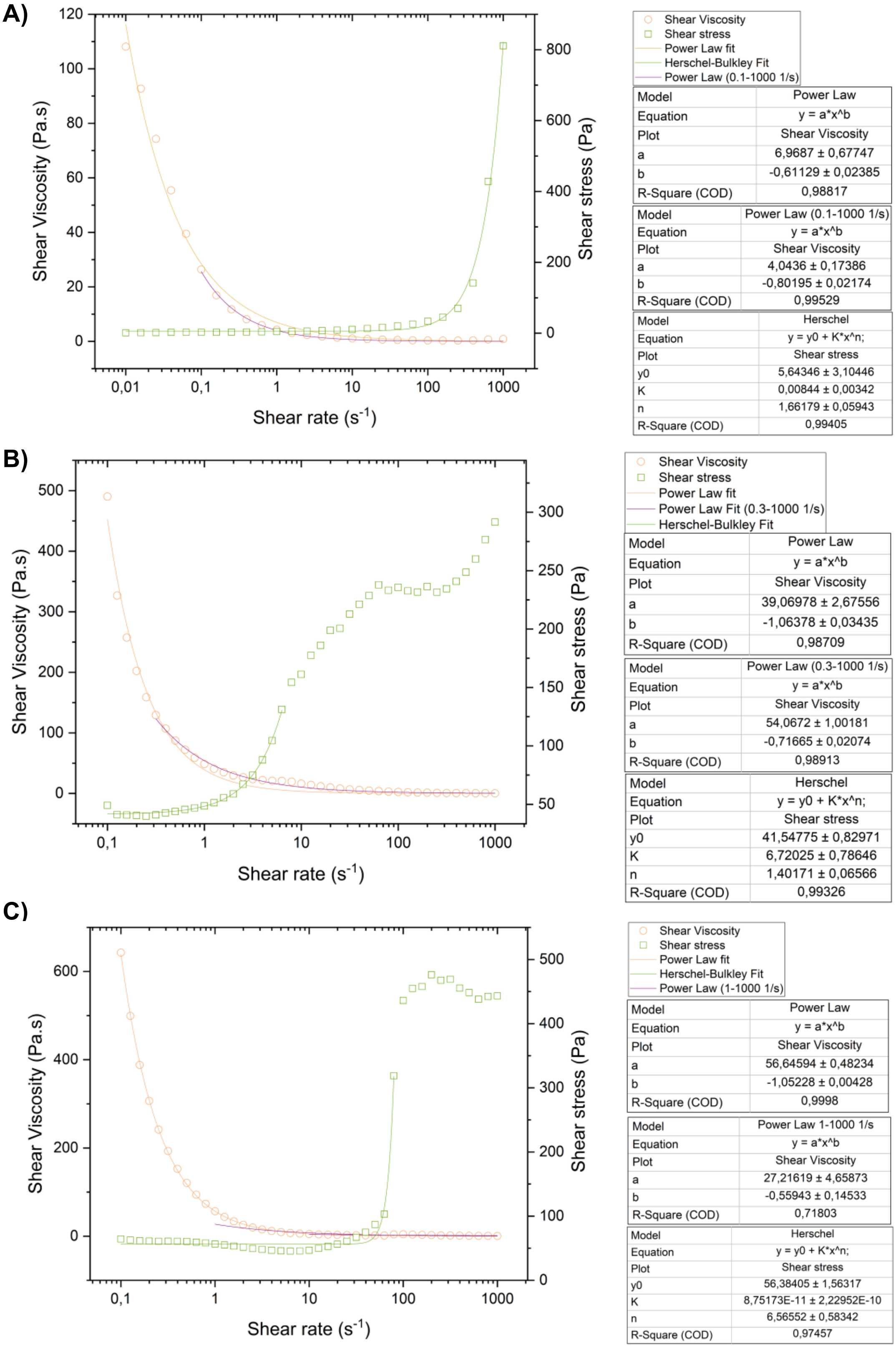

Figure S9: Fitting curves for A) LOW%, B) MEDIUM% and C) HIGH% PL-based inks. Power law was fit to the whole range of the curves (orange lines), but the obtained values

did not represent the reality of a shear thinning fluid, 0<*n*<1. This may be explained by the limitations of the model, previously described in [4,5]. This model fails to predict viscosity at very low shear rates, and also does not account for reduced viscosity near needle wall, which the Herschel-Bulkley model predicts more accurately. Therefore, Power Law was fit to a higher range of shear rates in all inks (purple lines), and Herschel-Bulkley was fit to lower ranges of shear rates (green lines) (n=3, mean values are represented)

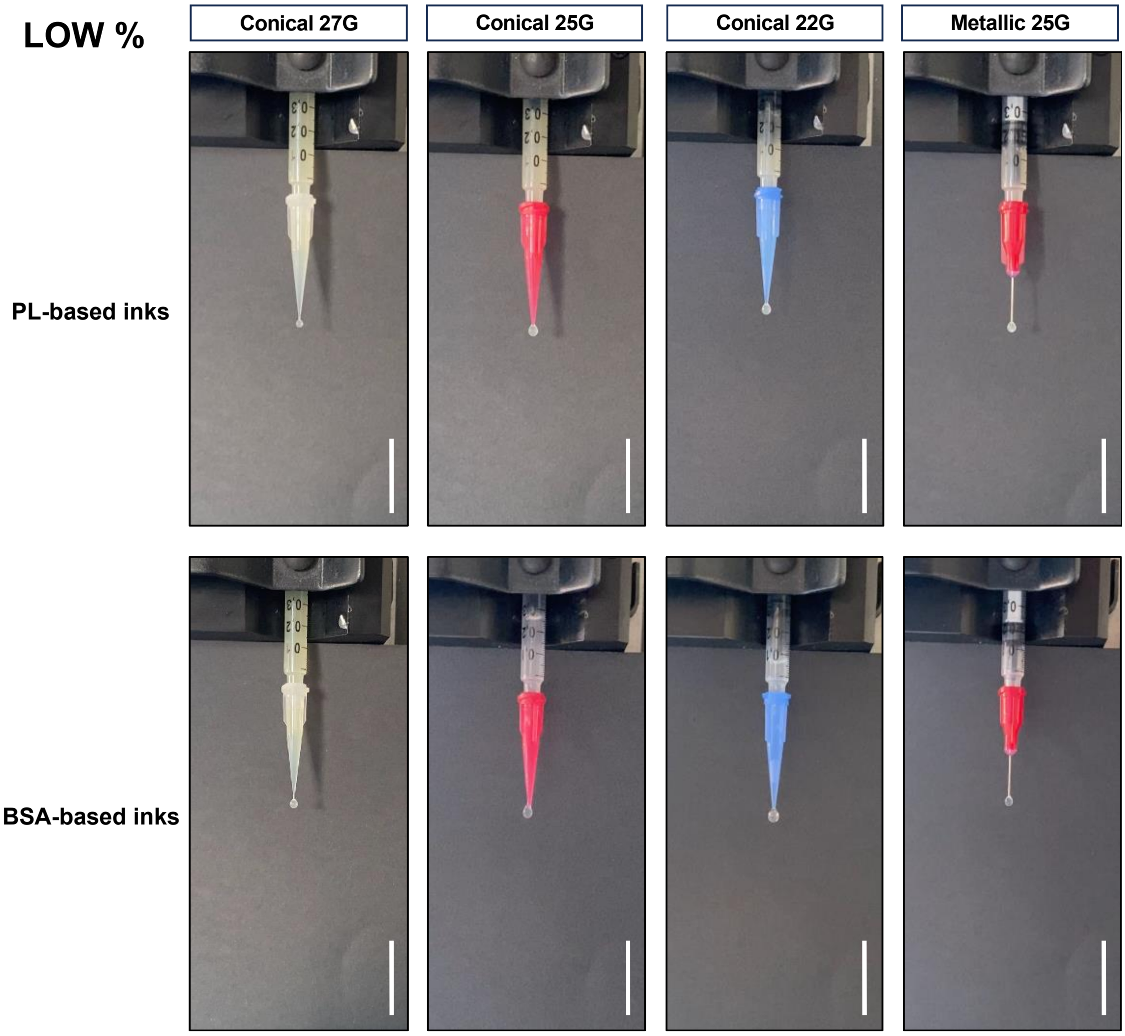

Figure S10: Extrudability of LOW% PL and BSA-based inks on 27G conical, 25G conical, 22G conical and 25G metallic needle. Scale bar: 2 cm

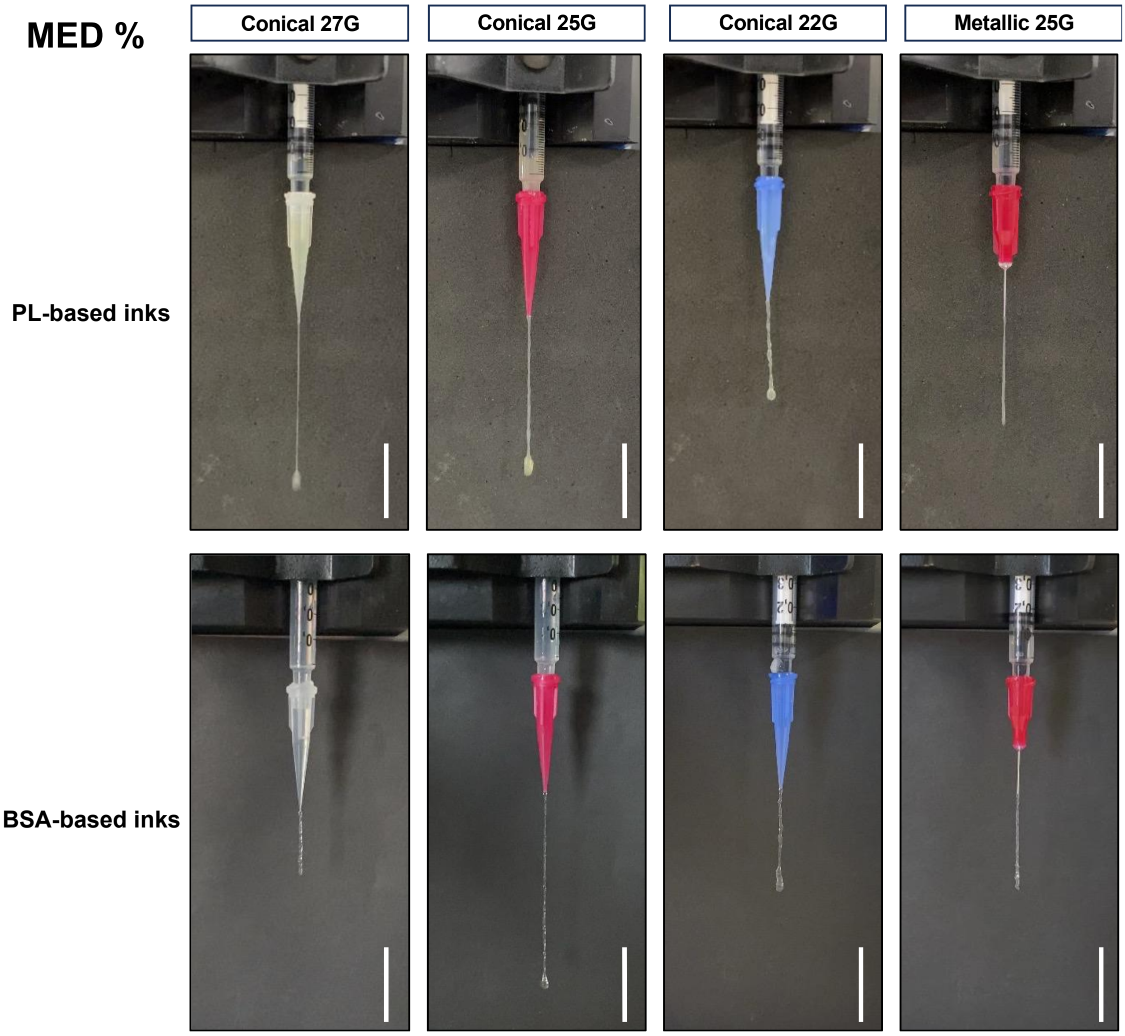

Figure S11: Extrudability of MEDIUM% PL and BSA-based inks on 27G conical, 25G conical, 22G conical and 25G metallic needle. Scale bar: 2cm.

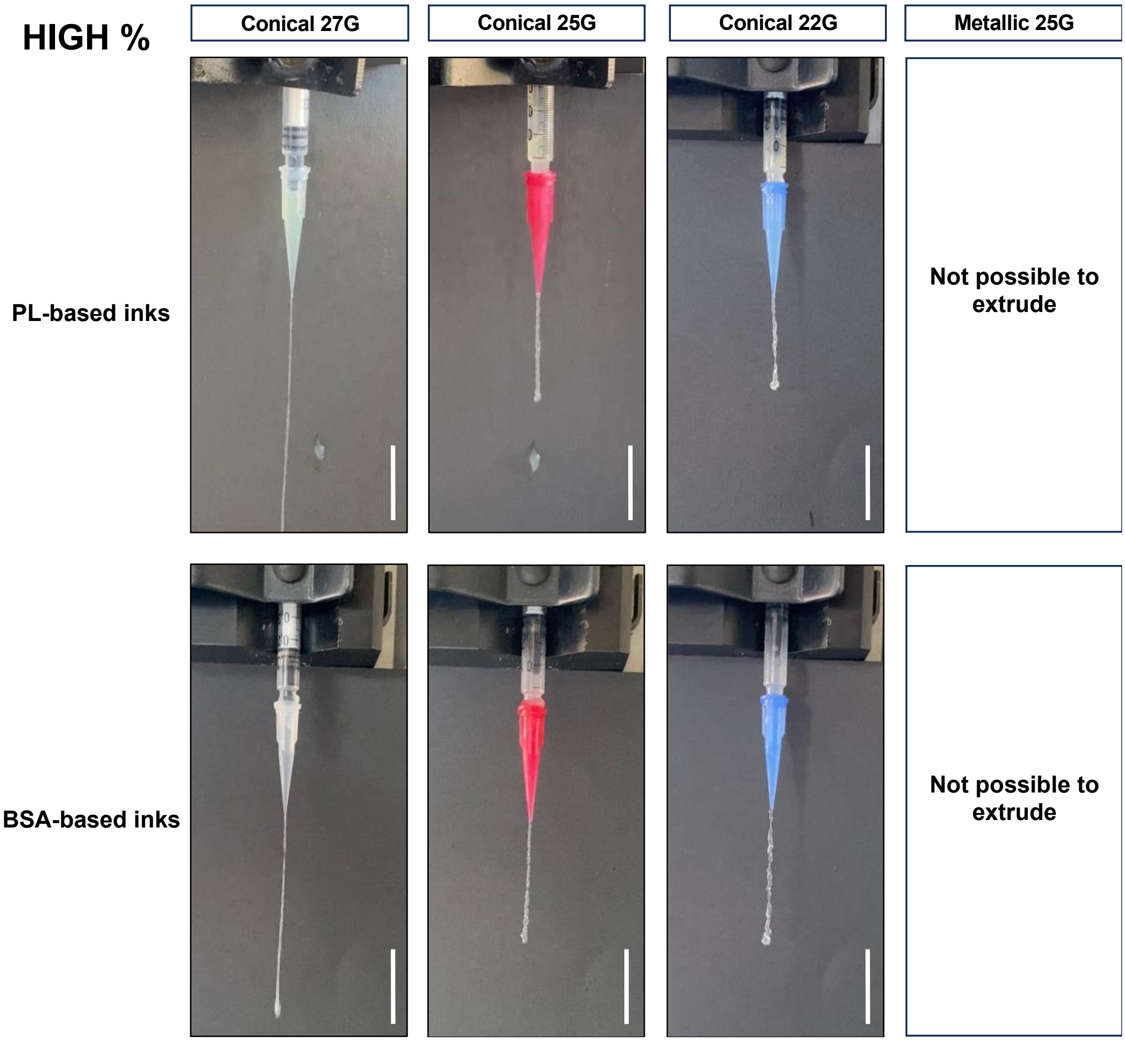

Figure S12: Extrudability of HIGH% PL and BSA-based inks on 27G conical, 25G conical, 22G conical and 25G metallic needle. Scale bar: 2 cm. It is to notice that using the 27G nozzle, these inks flowed quickly, and huge filaments were retrieved. Even though, because the applied extrusion printing is pneumatic, there was a lot of needle clogging during printing, especially when the filament retracted. We hypothesize that using a piston-driven dispensing type of printing head would allow the use of smaller nozzles and high accuracy in 3D printing using HIGH% inks; we also postulate that this would highly influence the yield stress and elastic recovery of these inks, so these assumptions are ought to be taken carefully.

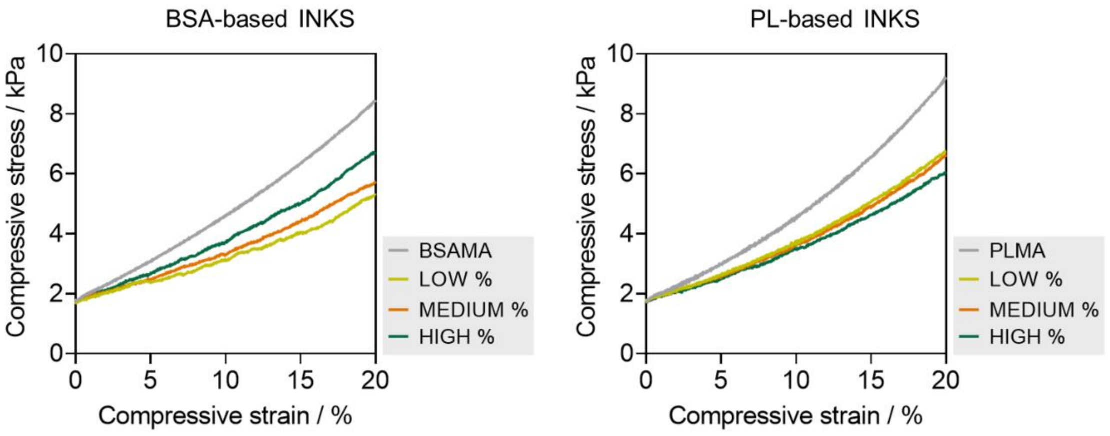

Figure S13: Representative stress/strain curves for BSA-based inks and PL-based inks hydrogels from 0 to 20% compressive strain.

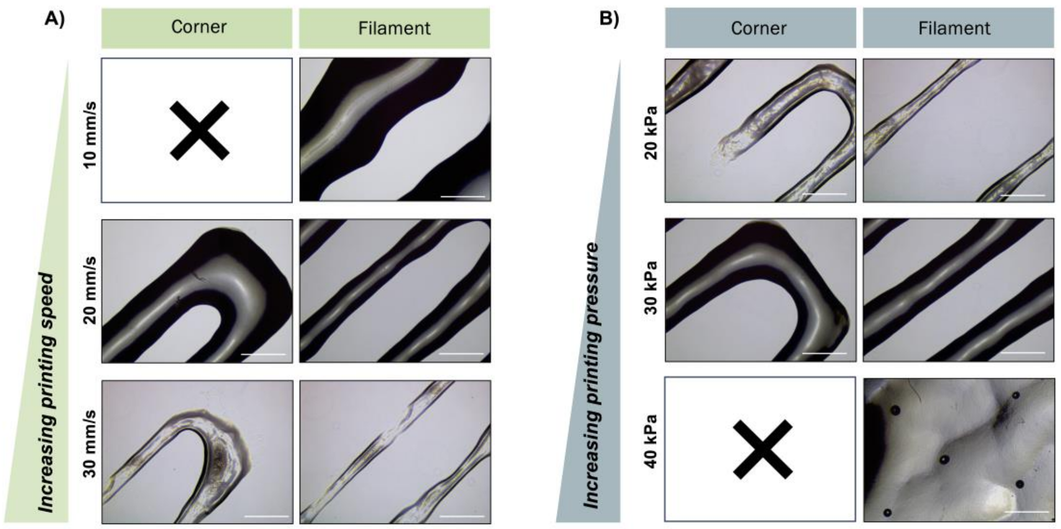

Figure S14: Representative micrographs of corner and filaments using BSA-based inks. A) Effect of printing speed; B) Effect of printing pressure. Missing images: since corners were not clearly defined after printing, micrographs were not acquired. Scale bar: 1mm.

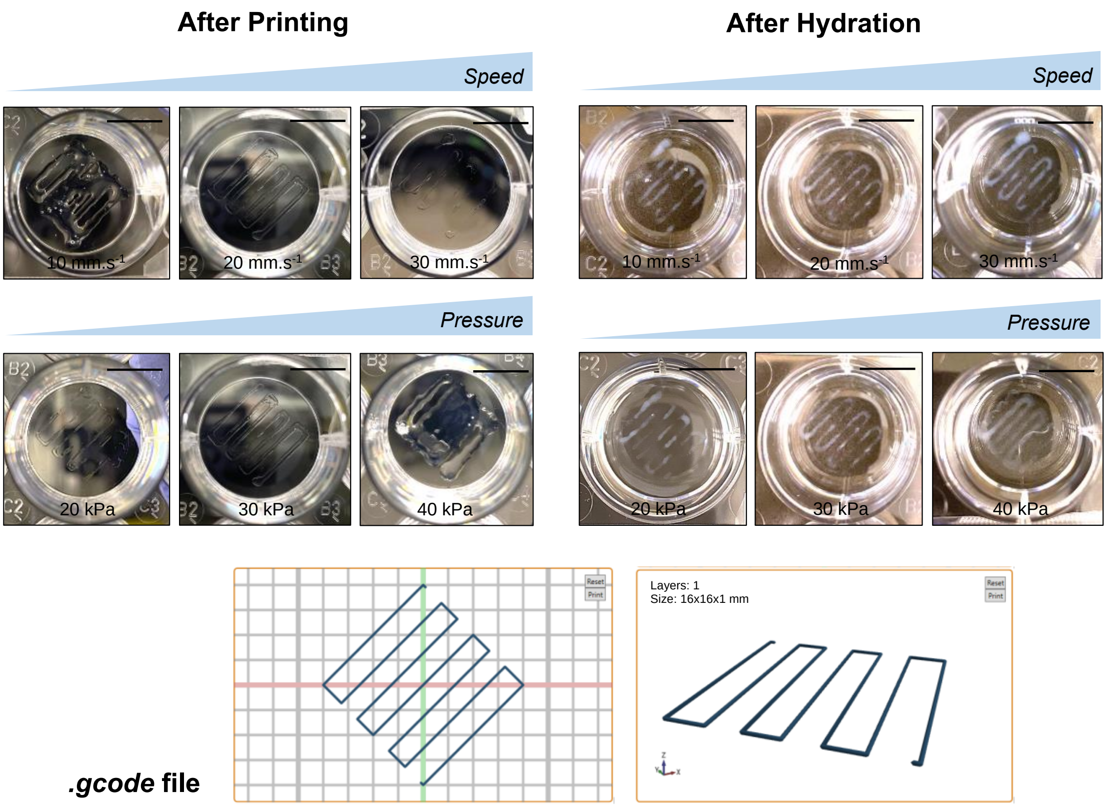

Figure S15: Visual aspect of the 1-layer design (.gcode file represented below) right after printing using PL-based inks on 12-well plates and after 24 hours of immersion in distilled water. Scale bar: 1 cm.

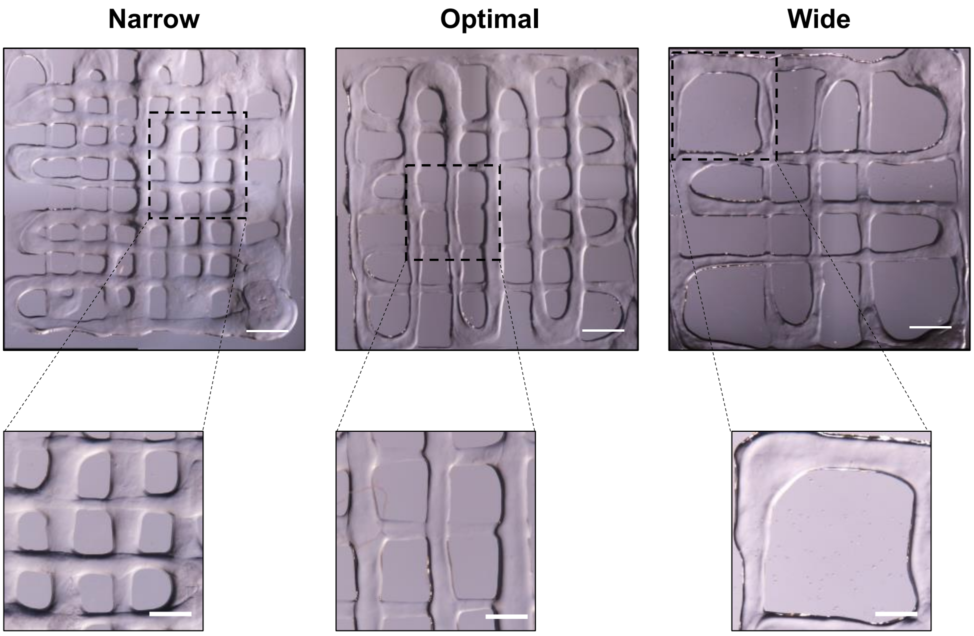

Figure S16: Grids obtained using BSA-based inks. Narrow, optimal and wide representing decreasing infill percentage. Scale bar: 2mm. Below: zoom-in, depicting pore geometry and size. Scale bar: 1mm.

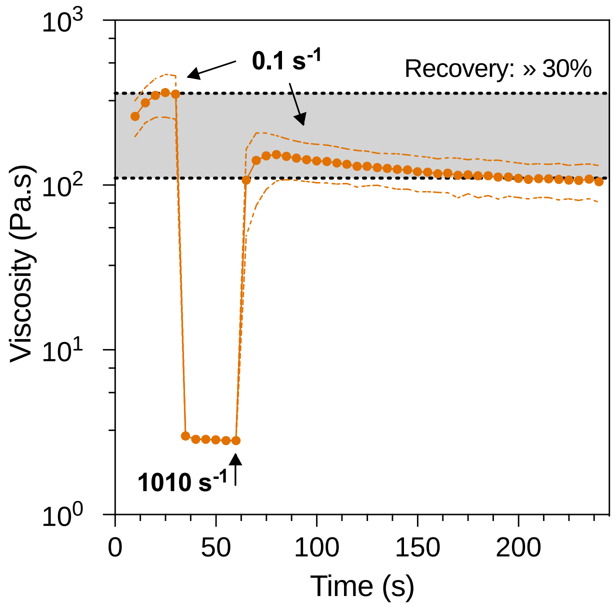

Figure S17: Three intervals thixotropy test (3ITT) performed to PL-based ink (MEDIUM%) to characterize thixotropic behavior of the inks when submitted to high shear rates. First interval: 0.1 s^{-1} during 30 seconds, second interval 1010 s^{-1} during 30 seconds, third interval: 0.1 s^{-1} during 180 seconds. The value of shear rate of the second interval was obtained from Table 1, as the calculated value of shear rate at the wall for MEDIUM% of PL-based inks, at the chosen printing pressure and speed. Viscosity recovery of the inks – instant and long term – was calculated to be approximately 30% (n=3).

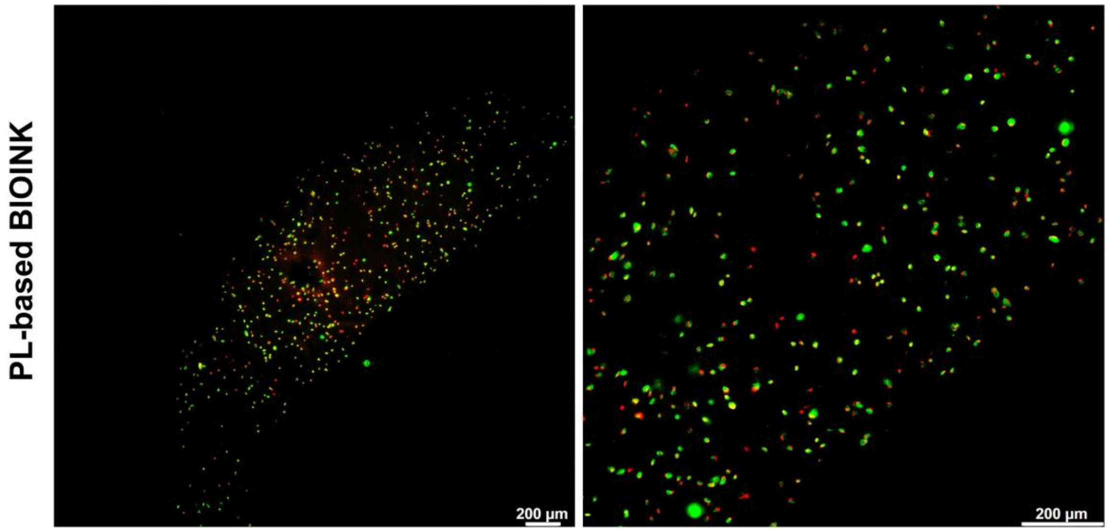

Figure S18: Representative live/dead images of PL-based bioink with human adipose stem cells (hASCs) after 24 hours in culture.

Supplementary Tables

Table S1: First trials performed to test the ratios of EDC to NHS to be used using only BSA protein. As indicated, different concentrations of BSA (10 and 20 wt%) were also tested. Gels were only formed when NHS concentration was equal or lower than EDC, obtaining liquid solutions when using 0.5:1 and 0.25:1 (EDC:NHS mass ratios). Also, when using 20 wt% BSA, gels were only viscous at a 0.43 wt% of EDC, which was very hard to control, given the low amount that needs to be added. Therefore, we opted using BSA at 10 wt% concentration for following trials.

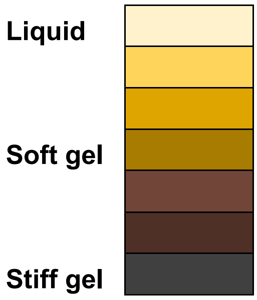

Concentration (wt%)		EDC:NHS mass ratio				
BSA Protein	EDC	1:0.25	1:0.5	1:1	0.5:1	0.25:1
20%	1,7%					
20%	0,85%					
20%	0,43%					
10%	0,85%					
10%	0,43%					

Table S2: Second trial performed using only BSAMA protein. Different buffers were tested (MES and PBS) and the reaction was monitored for 5h, in order to evaluate macroscopic changes in the hydrogels. that gels were formed after 15min independently of the buffer used. Nevertheless, after 5 hours or less, this reaction reverted, and the majority of the gels turned liquid.

Concentration (wv%)		Buffer (after 15min)		Buffer (after 5h)	
BSAMA Protein	EDC	MES	PBS	MES	PBS
10%	2,0%				
	3,5%				
	5,00%				

Table S3: First screening of double network formulations with respective control groups: BSA-MA+EDC (1), BSA-MA+EDC+NHS (2), BSA-MA+EDC+NHS+BSA-MA (3). These controls were compared to double network (DN) formulations, with different amounts of EDC/NHS (4, 5 and 6) and different initial protein concentrations (7 and 8) after 5 hours of reaction time.

	Concentrations (wt%)				Result
Condition	BSAMA	EDC	NHS (1:0.25)	DN	
1	10%	3,5%			
2	10%	3,5%	1,3%		
3	10%	3,5%	1,3%	10% BSA-MA	
4	10%	3,5%	0,87%	10% BSA	
5	10%	2,0%	0,5%	10% BSA	
6	10%	5,0%	1,25%	10% BSA	
7	15%	3,5%	1,3%	15% BSA	
8	20%	3,5%	1,3%	20% BSA	

Table S4: Predicted shear rate at the wall value for inks, using different gauge needles (18, 22, 25, 27) and different printing speeds (20, 10, 5 mm/s), for three flow indexes, given that *n*=1 corresponds to a Newtonian fluid, *n*=0.3 matches our ideal formulations and *n*=0.1 corresponds to a (theoretically) material with great shear-thinning properties.

Printing speed (mm/s)	Needle gauge	Shear rate (s^{-1}) (n=1)	Shear rate (s^{-1}) (n=0.3)	Shear rate (s^{-1}) (n=0.1)
20	27	761,90	1206,35	2476,19
20	25	615,38	974,36	2000
20	22	386,47	611,92	1256,04
20	18	190,93	302,31	620,53
10	27	380,95	603,17	1238,10
10	25	307,69	487,18	1000
10	22	193,24	305,96	628,02
10	18	95,47	151,15	310,26
5	27	190,48	301,59	619,05
5	25	153,85	243,59	500
5	22	93,62	152,98	314,01
5	18	47,73	75,58	155,13

References

[1] G. Ferracci, M. Zhu, M.S. Ibrahim, G. Ma, T.F. Fan, B.H. Lee, N.J. Cho, ACS Appl Bio Mater 3 (2020) 920–934.

[2] S.C. Santos, C.A. Custódio, J.F. Mano, Adv Healthc Mater 11 (2022) 2102383.

[3] C.R. Correia, R.P. Pirraco, M.T. Cerqueira, A.P. Marques, R.L. Reis, J.F. Mano, Scientific Reports 2016 6:1 6 (2016) 1–12.

[4] N. Paxton, W. Smolan, T. Böck, F. Melchels, J. Groll, T. Jungst, Biofabrication 9 (2017) 044107.

[5] A. Schwab, R. Levato, M. D'Este, S. Piluso, D. Eglin, J. Malda, Chem Rev 120 (2020) 11028–11055.
